# Network pharmacology-based approach to explore the underlying mechanism of sinomenine on sepsis-induced myocardial injury in rats

**DOI:** 10.3389/fphar.2023.1138858

**Published:** 2023-06-14

**Authors:** Linggang Sun, Zhiyun Chen, Yunjie Ni, Zhengfei He

**Affiliations:** Department of Cardiology, The First People’s Hospital of Fuyang Hangzhou, Hangzhou, Zhejiang, China

**Keywords:** sinomenine, sepsis, myocardial injury, network pharmacology, pathway

## Abstract

**Background:** Sepsis, a systemic disease, usually induces myocardial injury (MI), and sepsis-induced MI has become a significant contributor to sepsis-related deaths in the intensive care unit. The objective of this study is to investigate the role of sinomenine (SIN) on sepsis-induced MI and clarify the underlying mechanism based on the techniques of network pharmacology.

**Methods:** Cecum ligation and puncture (CLP) was adopted to induce sepsis in male Sprague-Dawley (SD) rats. Serum indicators, echocardiographic cardiac parameters, and hematoxylin and eosin (H&E) staining were conducted to gauge the severity of cardiac damage. The candidate targets and potential mechanism of SIN against sepsis-induced MI were analyzed via network pharmacology. Enzyme-linked immunosorbent assay was performed for detecting the serum concentration of inflammatory cytokines. Western blot was applied for evaluating the levels of protein expression. Terminal deoxynucleotidyl transferase-mediated dUTP biotin nick end labeling assay was applied to assess cardiomyocyte apoptosis.

**Results:** SIN significantly improved the cardiac functions, and attenuated myocardial structural damage of rats as compared with the CLP group. In total, 178 targets of SIN and 945 sepsis-related genes were identified, and 33 overlapped targets were considered as candidate targets of SIN against sepsis. Enrichment analysis results demonstrated that these putative targets were significantly associated with the Interleukin 17 (IL-17) signal pathway, inflammatory response, cytokines-mediated signal pathway, and Janus Kinase-Signal Transducers and Activators of Transcription (JAK-STAT) pathway. Molecular docking suggested that SIN had favorable binding affinities with Mitogen-Activated Protein Kinase 8 (MAPK8), Janus Kinase 1 (JAK1), Janus Kinase 2 (JAK2), Signal Transducer and Activator of Transcription 3 (STAT3), and nuclear factor kappa-B (NF-κB). SIN significantly reduced the serum concentration of Tumor Necrosis Factor-α (TNF-α), Interleukin 1 Beta (IL-1β), Interleukin 6 (IL-6), Interferon gamma (IFN-γ), and C-X-C Motif Chemokine Ligand 8 (CXCL8), lowered the protein expression of phosphorylated c-Jun N-terminal kinase 1 (JNK1), JAK1, JAK2, STAT3, NF-κB, and decreased the proportion of cleaved-caspase3/caspase3. In addition, SIN also significantly inhibited the apoptosis of cardiomyocytes as compared with the CLP group.

**Conclusion:** Based on network pharmacology analysis and corresponding experiments, it was concluded that SIN could mediate related targets and pathways to protect against sepsis-induced MI.

## 1 Introduction

Sepsis is a potentially fatal organ failure resulted from aberrant or dysfunctional host response to infection (Salomao et al., 2019). According to epidemiological investigations, 270,000 of the 1.7 million sepsis patients in the U.S. died in 2014, and sepsis has emerged as a prominent contributor to mortality among patients admitted to hospitals (McLaughlin et al., 2020). The incidence of sepsis is rising in China at a rate of 1.5% each year. Besides, the aging population and the wide application of invasive surgery contribute to the increase of morbidity and mortality of severe sepsis year by year (Corrales et al., 2022). Clinical and fundamental research showed that sepsis affects the cardiovascular system of patients, with myocardial injury (MI) occurring in about 40%–50% of them and leading to a death rate of 70%–90% (Xie et al., 2021). Sepsis-induced MI is first characterized by dysfunctions including decreased myocardial contractility and biventricular dilatation with decreased left ventricular ejection fraction (LVEF), followed by morphological changes such as myocardial cell degeneration, focal necrosis, and blurred myocardial striated lines. However, no targeted treatments are now available for sepsis-induced MI. Currently, multiple studies have linked the elevated inflammatory cytokines with sepsis-induced MI, and inflammation inhibition has been regarded as a promising therapeutic strategy for sepsis-induced MI.

Recently, the application of traditional Chinese Medicine (TCM) in treating sepsis-induced MI is attracting increasing attention. For instance, ShenFu injection showed favorable efficacy in treating sepsis-induced MI by reducing mitochondrial apoptosis (Xu et al., 2020). Sinomenine (SIN), as an alkaloid isolated from the root and stem of *Sinomenium acutum* (Thunb.) Rehder et Wilson or *S. acutum* var. cinereum., has been utilized extensively in the treatment of rheumatic diseases and arrhythmia (Liu et al., 2018). Accumulating evidence revealed that SIN exhibits diverse pharmacological effects, for instance, it is anti-inflammatory (Zeng and Tong, 2020), anti-cancer (Song L. et al., 2021), and analgesic (Jiang et al., 2020). Recently, SIN was reported to ameliorate lung injury in sepsis. The Nuclear factor erythroid 2-related factor 2-Kelch Like ECH Associated Protein 1 (Nrf2-Keap1) axis (Wang et al., 2020) or altering intestinal homeostasis through the aryl hydrocarbon receptor/Nrf2 axis (Song W. et al., 2021) are two potential mechanisms by which SIN could reduce septic acute lung damage in rats. Liu et al. claimed that SIN could improve lipopolysaccharide (LPS)-induced cardiomyocyte injury *in vitro* (Liu et al., 2021). Therefore, it is necessary to investigate the potential application and underlying mechanisms of SIN in treating sepsis-induced MI.

Network pharmacology has been developed by integrating biochemistry, bioinformatics, and system biology for studying the complex mechanism of TCM and discovering potential targets and mechanisms associated with various TCM monomers, such as artemisinin (Lin et al., 2021), melatonin (Song W. et al., 2021), and SIN (Li et al., 2021). In this study, the effects of SIN on cardiac dysfunctions was evaluated based on a rat model of sepsis. Network pharmacology was employed to identify SIN’s possible targets and pathways against sepsis-induced MI, and the binding affinity between SIN and corresponding candidate targets was simulated by molecular docking. Furthermore, the candidate targets and pathways were also validated *in vivo*.

## 2 Materials and methods

### 2.1 Prediction of SIN targets

The structure of SIN (CID: 5459308) was downloaded from the database of PubChem (https: https://pubchem.ncbi.nlm.nih.gov/). The candidate targets of SIN were predicted by the online tools of PharmMapper database (http://www.lilab-ecust.cn/pharmmapper/), Swiss Target Prediction (http://www.swisstargetprediction.ch/), HERB database (http://herb.ac.cn/), and TCM potential target database (TCM-PTD, http://tcm.zju.edu.cn/). After merging the results obtained from the four databases and excluding non-human genes, the rest genes were regarded as potential targets of SIN.

### 2.2 Screening sepsis-related genes

Sepsis-related genes were screened out from GeneCards (https://www.genecards.org/), Online Mendelian Inheritance in Man (OMIM, https://www.omim.org/), and Comparative Toxicogenomics Database (CTD, http://ctdbase.org/). “Sepsis” was used as a keyword for searching. After removing duplicates, the rest were identified as sepsis-related genes.

### 2.3 Network and enrichment analysis

The protein-protein interaction (PPI) of the shared targets between SIN and sepsis were retrieved from STRING (https://string-db.org/) with a medium confidence, and the PPI network was constructed with the use of Cytoscape software (https://cytoscape.org/). The topological parameters were calculated by the “Network Analyzer” plug-in. The “clusterprofiler” R package was used to analyze the enrichment of Gene Ontology (GO) and Kyoto Encyclopedia of Genes and Genomes (KEGG) pathways. The default threshold was set at a Bonferroni-corrected *p*-value of ≤0.05. The SIN-pathway-gene network was then established using Cytoscape software.

### 2.4 Molecular docking

Molecular docking analysis of SIN and its related targets was conducted with the use of AutoDock Vina software (version 1.1.2) to anticipate the strength of their interaction. The 2D structure of SIN was obtained from PubChem. The crystal structure of SIN-targets was obtained from RCSB protein data bank (RCSB PDB: https://www.rcsb.org/), including Mitogen-Activated Protein Kinase 8 (MAPK8, 3elj), nuclear factor kappa-B (NF-κB, 7RG5), Janus Kinase 1 (JAK1, 4ei4), Janus Kinase 2 (JAK2, 7f7w), and Signal Transducer And Activator of Transcription 3 (STAT3, 6nuq). Before the docking, PyMoL (version 4.5.0) software was used for protein preparation by removing water molecules, solvent molecules, and other protein chains. Then, the software of AutoDock Tools 1.5.6 was used to add nonpolar hydrogens and calculate Gasteiger charges of protein structures. The Lamarckian Genetic algorithm was used to perform the conformational search and generate 100 conformations. The conformation with the best affinity was selected as the final docking conformation. The 2D diagrams of the SIN-targets complex were generated using LigPlus (version 2.24), and the 3D complex was visualized by PyMoL.

### 2.5 Animal model and SIN treatment

The Institutional Animal Care and Use Committee of Zhejiang Center of Laboratory Animals approved the animal procedures and experimental protocols (Approval Number: ZJCLA-IACUC-20020101). Male Sprague-Dawley (SD) rats (180–200 g) were obtained from Hangzhou Medical College Laboratory Animal Center, and maintained in a specified pathogen-free environment with unlimited availability of food and water on a 12-h day and night cycle. The sepsis model was induced by cecum ligation and puncture (CLP), following the previously described procedures (Mishra and Choudhury, 2018). The four groups—sham, CLP, CLP + LSIN, and CLP + HSIN groups—each containing ten experimental rats, were randomly allocated. The exposed cecum of the rats was sutured with 3–0 silk suture 1.2 cm to its distal end and punctured twice with a 22-gauge needle to create sepsis model. Following the surgical procedure, all rats received a subcutaneous injection of 50 mL/kg compound sodium chloride. The rats in the sham group underwent identical procedures as described above, excluding the CLP treatment. In the CLP + LSIN and CLP + HSIN groups, rats received SIN administration via tail vein injection at doses of 50 mg/kg and 100 mg/kg, respectively, 15 min prior to sepsis induction. After 24 h of sepsis, the hearts were extracted and a total of 500 mL blood was collected to obtain serum.

### 2.6 Determination of serum biochemical parameters

After blood collection from each group of rats, serum obtained by subjecting the blood samples to centrifugation at 3,000 *g*/min for 30 min. An automated analyzer (Modular DPP H7600; Roche Diagnostics, Basel, Switzerland) was employed to assess the serum concentration of lactate dehydrogenase (LDH) and creatine kinase and its MB isoenzyme (CK-MB). Enzyme linked immunosorbent assay (ELISA)-based assay was performed for the concentration detection of cardiac troponin I (cTnI, mlbio, China, Shanghai), cardiac myosin light chain-1 (cMLC1, EK-Bioscience, China, Shanghai), as well as several inflammatory cytokines such as Tumor Necrosis Factor-α (TNF-α, Applygen, China, Beijing), Interleukin 1 Beta (IL-1β, wksubio, China, Shanghai), Interleukin 6 (IL-6, Thermo Fisher Scientific, USA, Massachusetts), C-X-C Motif Chemokine Ligand 8 (CXCL8, Shanghai yiyan bio-technology Co. Ltd., China, Shanghai), and Interferon gamma (IFN-γ, Sino Biological, China, Beijing) in plasma.

### 2.7 Echocardiography

The cardiac parameters were evaluated after 12 h of CLP by echocardiography according to the methodology reported previously (Bayer et al., 2021). The rats were anesthetized with a mixture of 2% isoflurane and 0.5 L/min 100% O_2_ before they were positioned on a warming pad (37 °C). A Vevo 2,100 Imaging System was used to take echocardiographic measures (FUJIFILM VisualSonics, Inc., Toronto, Ontario, Canada). To evaluate heart functioning, the ejection fraction (EF) and left ventricular interior dimension (LVID) were calculated. All measurements were conducted by a cardiologist who was unaware of the experimental details, ensuring a blinded assessment.

### 2.8 Hematoxylin and eosin (H&E) staining

The rat myocardial tissues were collected from every group 24 h after CLP and immediately fixed with 4% paraformaldehyde overnight at room temperature to facilitate subsequent histological analysis. The materials were then divided into 4 μm-thick slices and embedded in paraffin. After that, the slices underwent H&E staining and onserved under a light microscope at a magnification of ×400.

### 2.9 Western blot

The myocardial tissues were homogenized using Radio Immunoprecipitation Assay (RIPA, Thermo Fisher Scientific, USA, Massachusetts) lysis buffer, and subsequently centrifuged at 13,200 g at 4°C for 30 min. To determine the protein concentrations, the Bradford assay was conducted, and the supernatant was collected for total protein analysis. Subsequently, the extracted proteins (25 μg) were subjected to Sodium dodecyl sulfate-polyacrylamide gel electrophoresis (SDS-PAGE) for seperation, blotted and probed with the following antibodies: anti-c-Jun N-terminal kinase 1 (JNK1, ab199380, Abcam, UK, Cambridge, 1/2,500), anti-phospho-JNK1 (ab215208, Abcam, UK, Cambridge, 1/1,000), anti-NF-κB (ab16502, Abcam, UK, Cambridge, 1/1,000), anti-phospho-NF-κB (ab76302, Abcam, UK, Cambridge, 1/1,000), anti-JAK1 (ab133666, Abcam, UK, Cambridge, 1/1,000); anti-phospho-JAK1 (ab215338, Abcam, UK, Cambridge, 1/5,000); anti-JAK2 (ab108596, Abcam, UK, Cambridge, 1/1,000); anti-phospho-JAK2 (ab32101, Abcam, UK, Cambridge, 1/1,000); anti-STAT3 (ab68153, Abcam, UK, Cambridge, 1/2000); anti-phospho-STAT3 (ab76315, Abcam, UK, Cambridge, 1/1,000); anti-Caspase-3 (9662S, Cell Signaling Technology, USA, Massachusetts 1/1,000); anti-Cleaved Caspase-3 (9664S, Cell Signaling Technology, USA, Massachusetts). For loading control, the blots were probed with antibody for GAPDH (ab8245, Abcam, UK, Cambridge, 1/500). The blots were measured by the chemiluminescence system (Millipore, Billerica, MA, USA), and the signals were quantified by densitometry. Use ImageJ 1.8.0 (https://imagej.nih.gov/ij/) to read the density of the bands.

### 2.10 Terminal deoxynucleotidyl transferase dUTP nick end labeling (TUNEL) staining

TUNEL test was used to ascertain the rate of cardiomyocyte apoptosis in the heart tissues of rats according to relevant instructions (Roche, USA). The heart tissue sections were fixed and permeated, followed by co-staining of TUNEL and 4′,6-diamidino-2-phenylindole (DAPI).

### 2.11 Statistical analysis

Statistical analyses were performed using SPSS 25.0 software, and the results were reported as the mean ± standard deviation. A *p*-value ≤0.05 is considered to be statistically significant.

## 3 Results

### 3.1 SIN improved the cardiac function of septic rats

**FIGURE 1:**
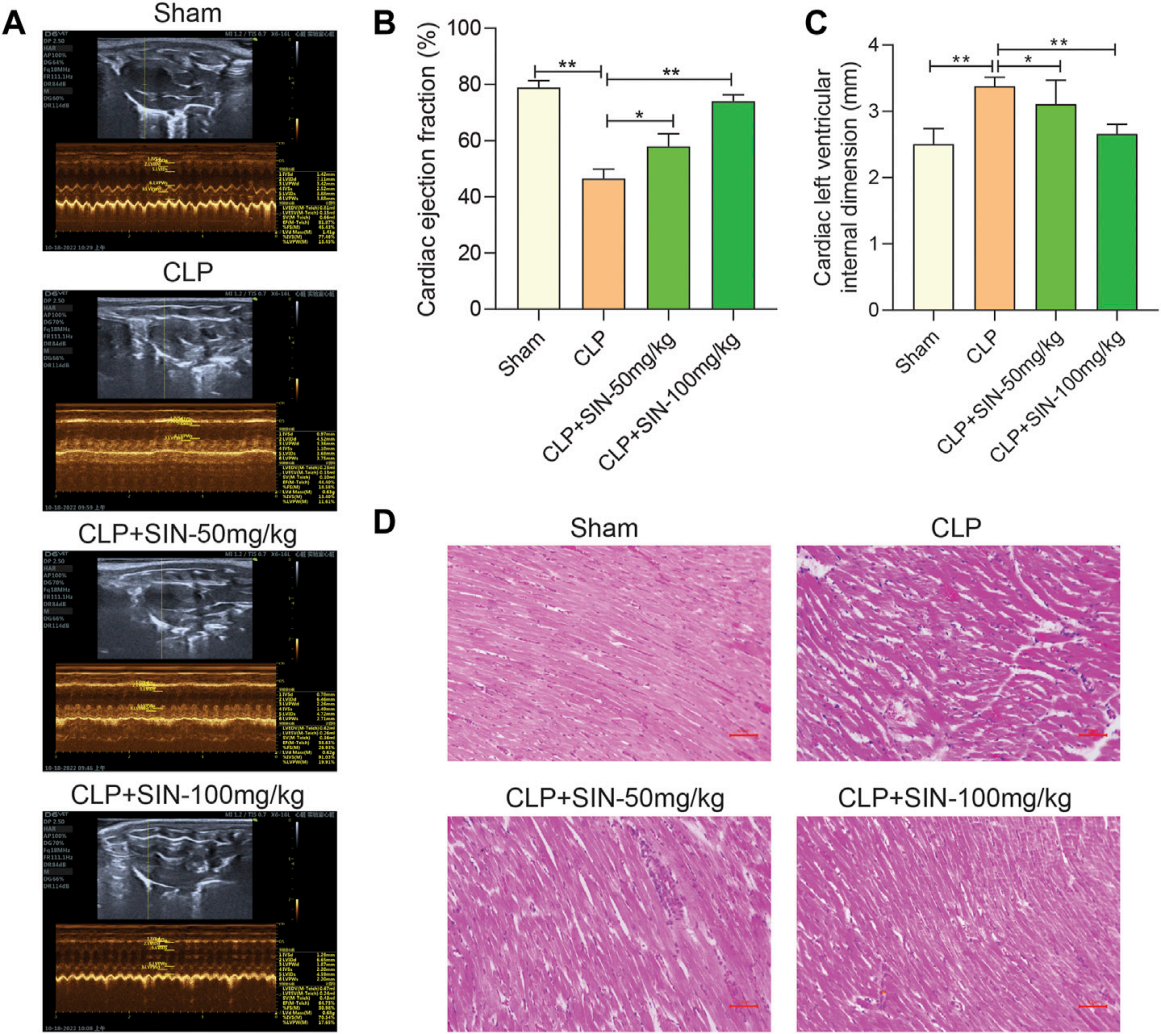
SIN increased the survival of rats with sepsis brought on by CLP and reduced the severity of the cardiac dysfunction those animals experienced. **(A)** Representative images of echocardiography for each group. Ejection fraction **(B)** and left ventricular internal dimension **(C)** were assessed using echocardiography as indicators of cardiac function. **(D)** Rat cardiac tissues’ histological alterations at 12 h after CLP (H&E staining, ×40). *n* = 6. *p < 0.05, **p < 0.01.

To determine whether SIN has cardioprotective effects, the rats that developed sepsis via CLP were examined using echocardiography. SIN demonstrated a dose-dependent effect in significantly increasing the low EF in rats with CLP-induced sepsis, whereas significantly reduced the elevated LVID of septic rats in the CLP group. These findings suggested that SIN ameliorated cardiac function in septic rats (Figures 1A–C). Moreover, histopathological changes in the rat myocardial tissues were observed via H&E staining to assess the beneficial effect of SIN on MI in septic rats. As shown in Figure 1D, there was no degeneration, necrosis, or aberrant alterations in the myocardial interstitium of rats in the sham group, although they did have obvious transverse stripes of myocardial fibers. Rats in the CLP group showed significant pathological changes in their myocardial tissues, including myocardial fiber partial rupture and breakdown, myocardial stripe blur partial disappearance, and interstitial edema. Notably, administration of 50 and 100 mg/kg SIN considerably reduced these pathogenic alterations.

### 3.2 SIN attenuated the myocardial injuries of septic rats

**FIGURE 2:**
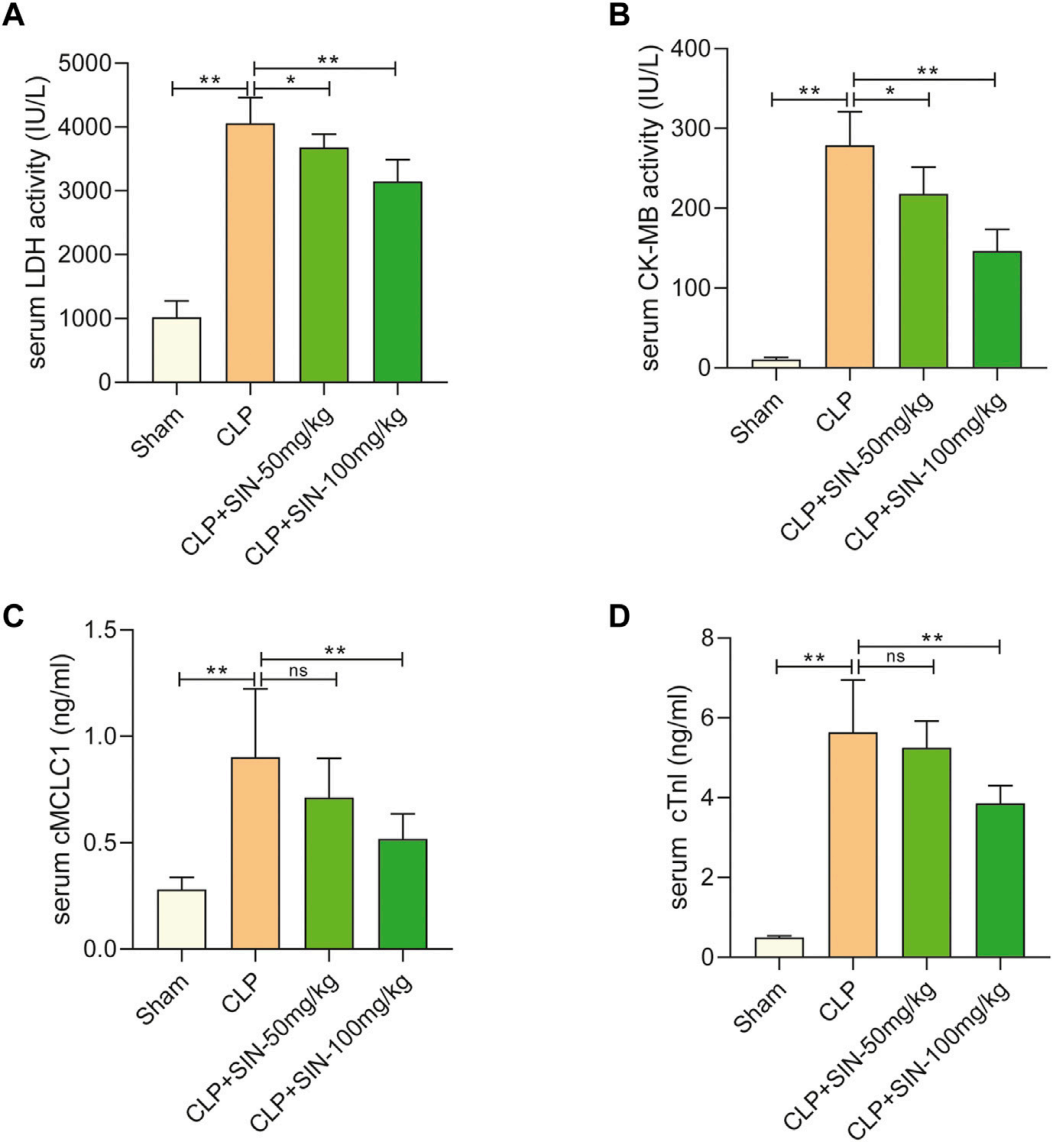
SIN reduced the cardiac dysfunction brought on by CLP. **(A)** Rat serum LDH response to SIN. **(B)** Rat serum CK-MB response to SIN. **(C)** Rat serum cMLC1 response to SIN. **(D)** Rat serum cTnI response to SIN. *n* = 6. *p < 0.05, **p < 0.01.

Four serum biomarkers were used in this study for evaluation of the myocardial injuries of rats: LDH, CK-MB, cMLC1, and cTnI (Shiroorkar et al., 2020). Twelve hours after CLP surgery, the concentrations of LDH, CK-MB, cTnI, and cMLC1 in the serum were measured in each group. As shown in Figure 2, the concentration of LDH, CK-MB, cMLC1, and cTnI in the serum of septic rats was obviously elevated as compared with the normal rats, indicating the presence of CLP-induced MI. Importantly, SIN demonstrated a dose-dependent effect in significantly decreasing the aforementioned injury in rats with CLP-induced sepsis. These data suggested that SIN can attenuate MI and ameliorate myocardial function.

### 3.3 Potential targets and pathways of SIN against sepsis-induced MI

**FIGURE 3:**
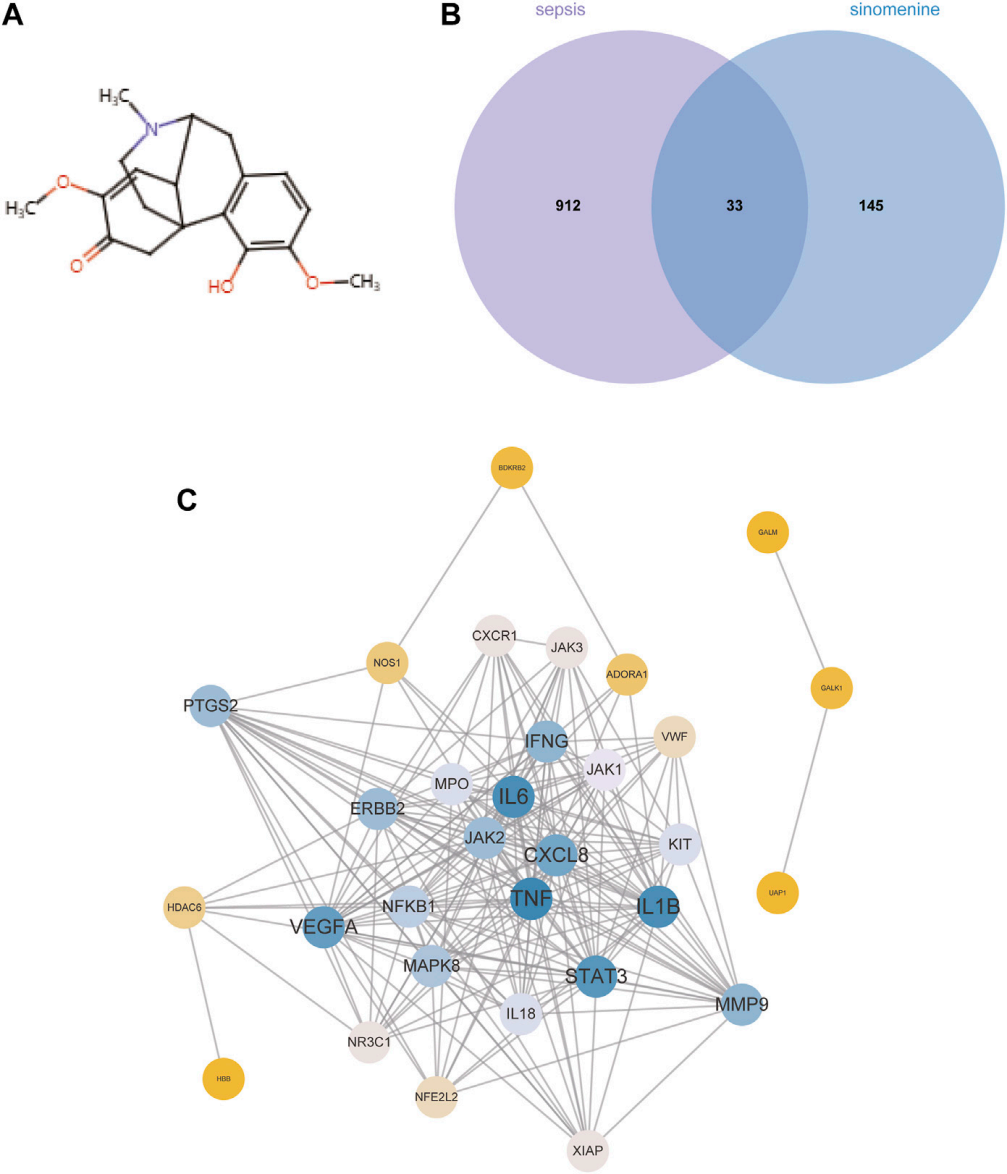
The candidate targets of SIN against sepsis-induced MI. **(A)** The structure of SIN. **(B)** Venn diagram of the genes associated with sepsis and SIN targets. **(C)** The PPI network of the 33 candidate targets was generated by Cytoscape.

Figure 3A depicts the chemical structure of SIN**.** In total, 178 targets of SIN were identified (Supplementary Table S1), including 55 in SwissTargetPrediction, 103 in PharmMapper, 16 in HERB, and 10 in TCM-PTD. Meanwhile, 945 sepsis-related genes were screened out from GeneCard (902), OMIM (77), and Therapeutic Target Database (TTD) (67) (Supplementary Table S2). There were 33 shared targets between the targets of SIN and sepsis-related genes, as shown in Figure 3B. These proteins were identified as candidate targets of SIN against sepsis. Figure 3C shows the PPI network of these common targets, and the top 4 nodes with a greater degree were TNF, IL6, IL1β, and STAT3, respectively. These data indicated that the four targets play more crucial roles in the treatment of sepsis-induced MI by SIN.

**FIGURE 4:**
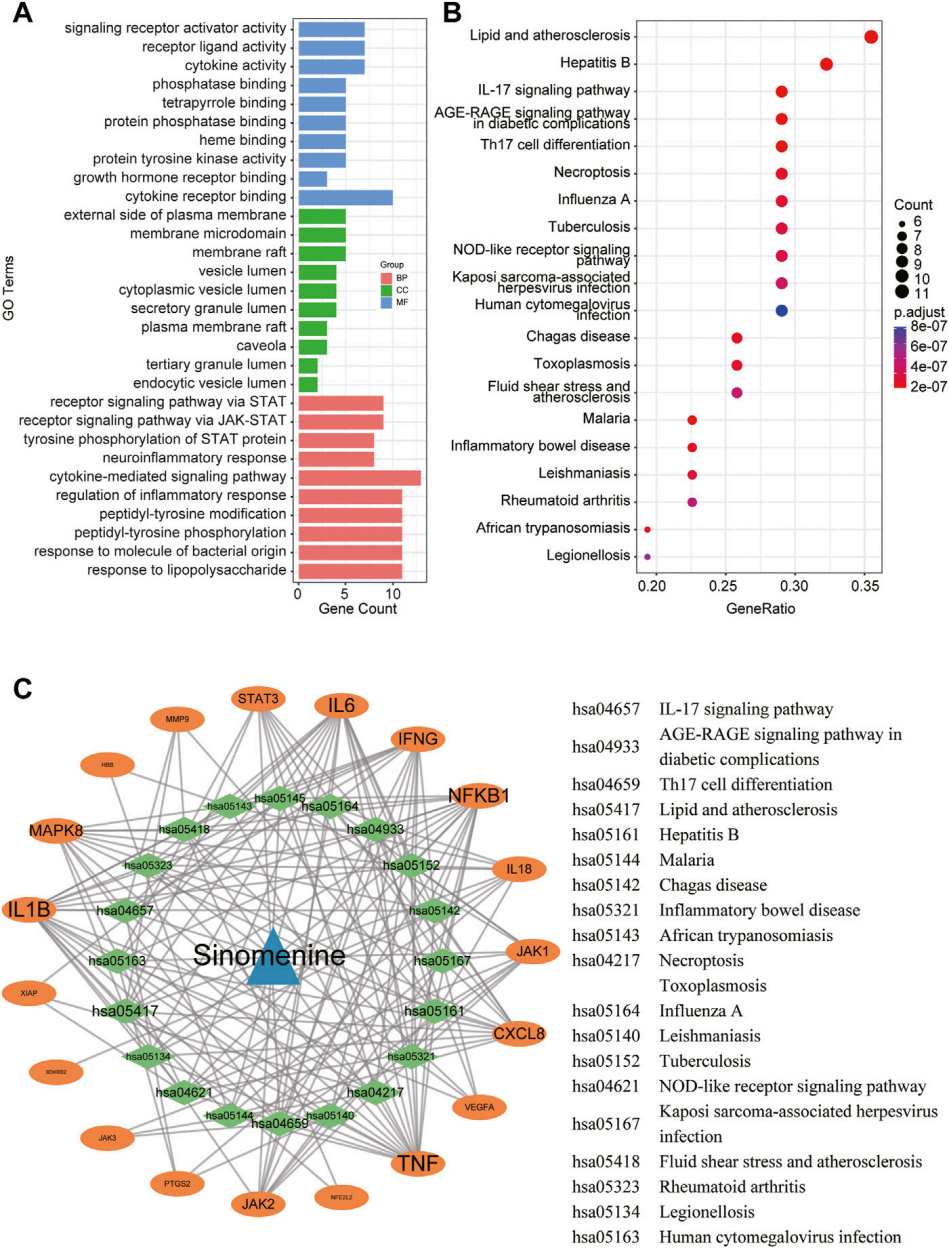
A network pharmacology approach was used to determine the fundamental mechanisms of SIN against sepsis. **(A)** The top 10 terms of GO analysis of the candidate targets of SIN against sepsis-induced MI. CC: cellular component, BP: biological process, MF: molecular function. **(B)** The top 20 pathways of KEGG pathway enrichment analysis that ranked by gene count. **(C)** The SIN-targets-pathway network showed detailed interactions between the hub targets and pathways. Orange cycles stand in for hub targets, while green circles represent the top 20 pathways that SIN uses to combat sepsis.

To reveal the potential functions and pathways of the candidate targets, functional enrichment analyses were conducted accordingly, and the findings are summarized in Supplementary Tables S3, S4. Figure 4A displays the top 10 GO terms for biological process, cellular component, and molecular function. It was demonstrated that the candidate targets were primarily associated with cytokines mediation, inflammatory response, and phosphorylation of STAT protein. The enriched KEGG pathways primarily included Interleukin 17 (IL-17) signaling pathway, nucleotide-binding oligomerization domain (NOD)-like receptor signaling pathway, as well as various pathways related to viral or inflammatory diseases (Figure 4B). To better understand the correlations between candidate targets and enriched pathways, a gene-pathway network was established. Figure 4C illustrates that the larger the node font is, the more pathways the corresponding target are involved in.

### 3.4 Binding affinity of SIN with target proteins

**FIGURE 5:**
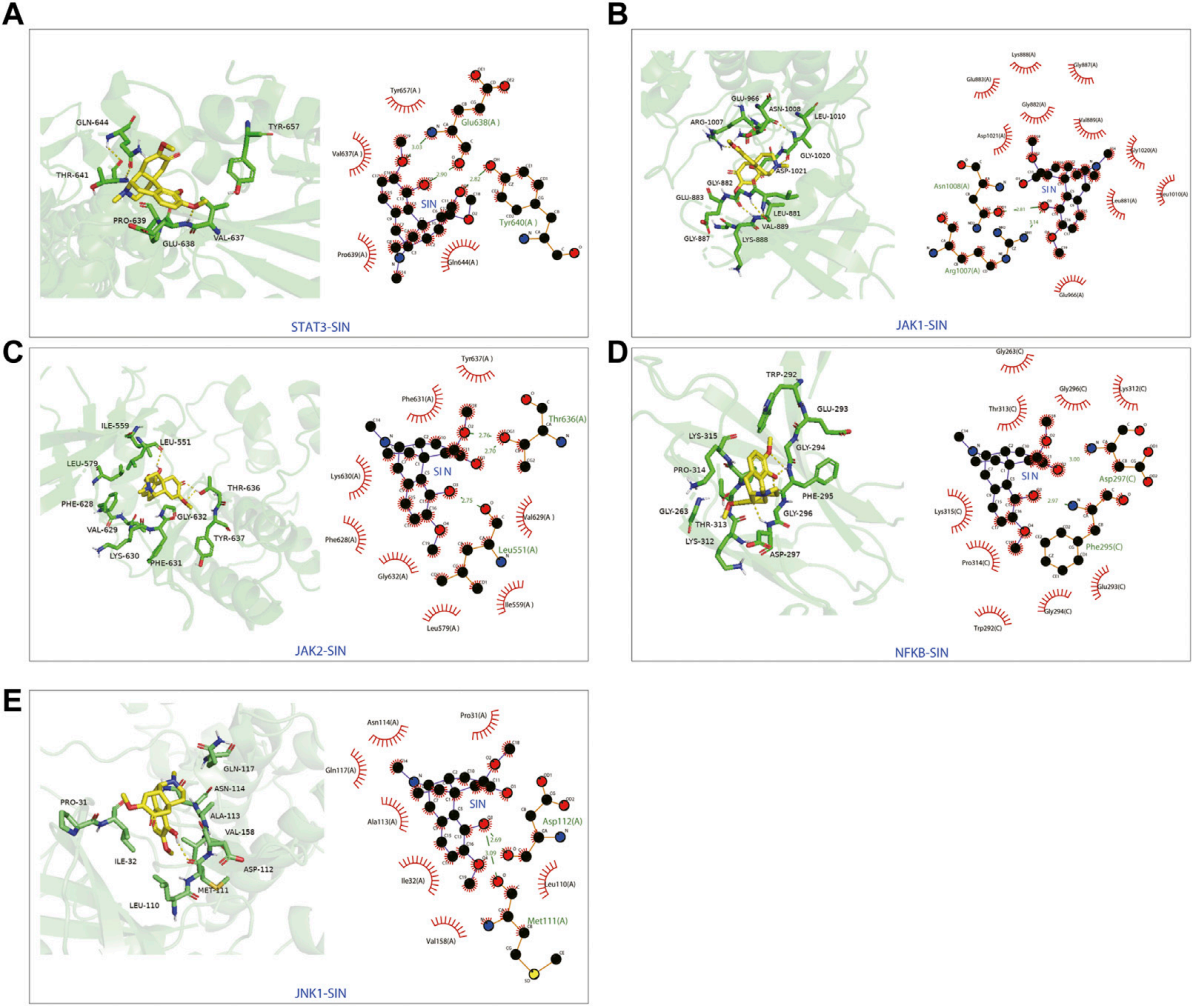
The 3D and 2D diagrams showing the molecular docking pose of SIN with **(A)** STAT3, **(B)** JAK1, **(C)** JAK2, **(D)** NF-κB, and **(E)** JNK1.

The candidate targets’ possible binding modes with SIN were evaluated using molecular docking analysis (Figure 5). The autodock scores were summarized in Table 1, and a lower score indicated a better binding affinity between SIN and the proteins. Our data indicated that SIN was most tightly bound to JAK1 and loosely bound to JNK1. Additionally, hydrogen bonds were observed in all SIN-target complexes and the O3 in SIN was prone to form hydrogen bonds with residues. The main groups that bind residues with H donor moieties at the terminal are believed to be the carbonyl, methoxy, and hydroxy groups. The 2D and 3D docking images showed that SIN interacted with JAK1, JAK2, STAT3, JNK1, and NF-κB via hydrogen bonds and hydrophobic contacts. These data indicated that SIN can interact with JAK1, JAK2, STAT3, JNK1, and NF-κB to form compact complexes. Therefore, the influence of SIN on the protein expression of these targets was further validated *in vivo*.

**TABLE 1:** The autodock score and hydrogen bonds of putative targets with sinomenine from molecular docking analysis.

Genes	PDB accession number	Autodock score (kcal/mol)	Hydrogen bonds
JAK1	4ei4	−6.23	Asn1008(OD1):SIN(O3)
			Arg1007(NH1):SIN(O3)
JAK2	7f7w	−5.54	Thr636(OG1):SIN(O2)
			Thr636(OG1):SIN(O1)
			Leu551O):SIN(O3)
JNK1	3elj	−5.25	Asp112O):SIN(O3)
			Met111O):SIN(O3)
NF-κB	1ikn	−6.06	Asp297N):SIN(O1)
			Phe295N):SIN(O3)
STAT3	6nuq	−5.59	Glu638N):SIN(O4)
			Tye640(OH):SIN(O1)
			Glu638O):SIN(O4)

Abbreviation: SIN, sinomenine; PDB, protein data bank.

### 3.5 SIN regulated the inflammatory response via JNK/NF-κB pathway

**FIGURE 6:**
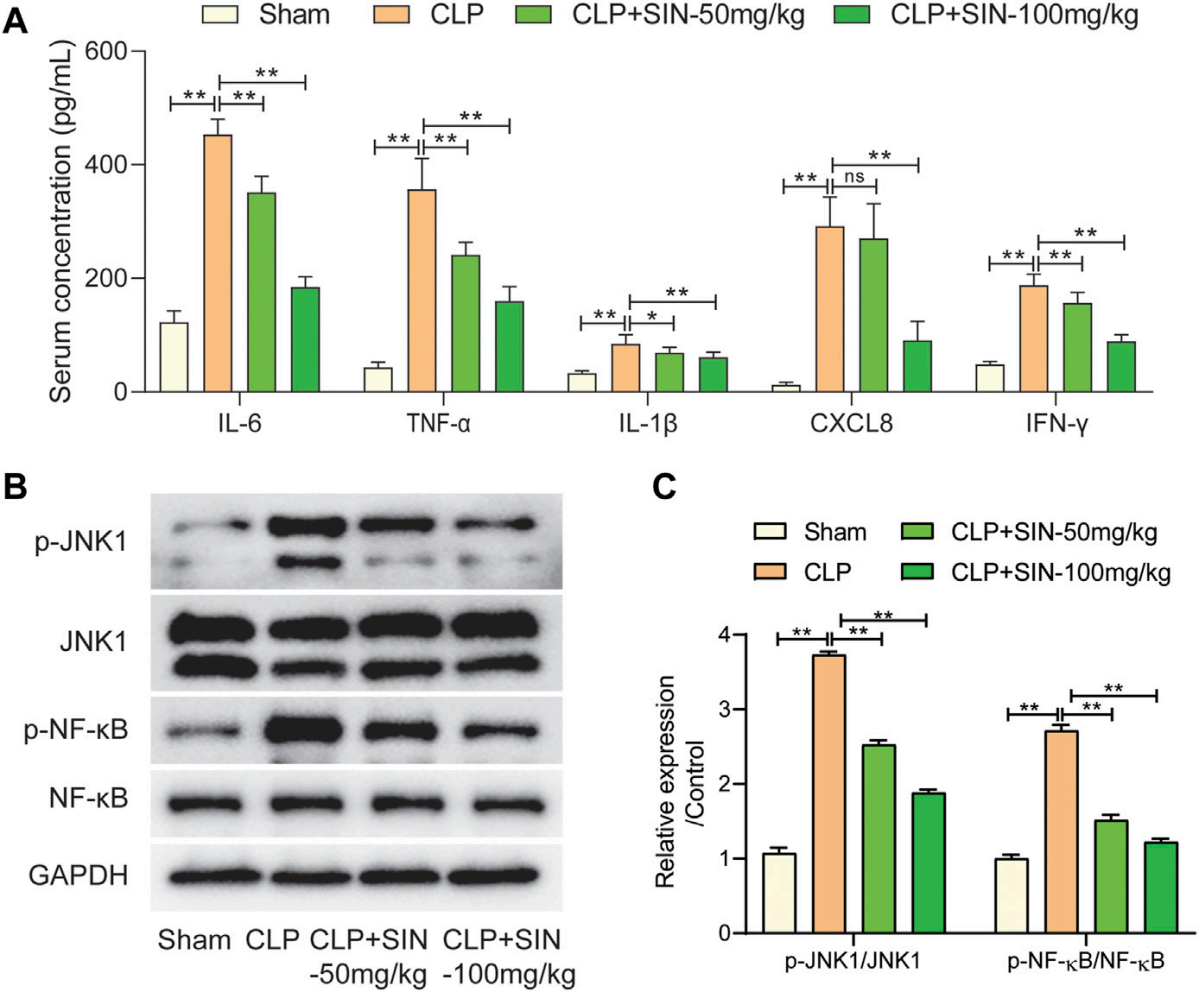
The regulation of SIN on the targets in the IL-17 signal pathway. **(A)** Comparison of serum concentration of inflammatory cytokines in each group. **(B,C)** The expression levels of JNK1/NF-κB and phosphorylated JNK1/NF-κB were examined. *n* = 6. *p < 0.05, **p < 0.01.

The IL-17 pathway was found to be implicated in the effects of SIN on mitigating sepsis-induced cardiac dysfunction in this study via network pharmacology and KEGG enrichment analysis. The targets of SIN involved in the IL-17 signal pathway, including JNK1, NF-κB, IL-1β, *etc.*, were found and illustrated in Figure 4C. Therefore, the effect of SIN on these targets were detected *in vivo*. As shown in Figure 6A, SIN demonstrated a significant dose-dependent effect in significantly reversing the increased inflammatory cytokines induced by CLP, including TNF-α, IL-1β, IL-6, IFN-γ, and CXCL8. Based on molecular docking simulations, SIN was found to exhibit binding affinity with JNK1 and NF-κB, and may have potential impacts on the phosphorylation of these targets. The expression levels of p-JNK1 and p-NF-κB were shown to be significantly elevated by CLP, but reduced after treatment with SIN (Figure 6B). Taken together, these findings implied that the IL-17 signal pathway is expected to attenuate the effect of SIN on sepsis-induced MI.

### 3.6 SIN decreased cardiomyocyte apoptosis by regulating JAK/STAT signal pathway

**FIGURE 7:**
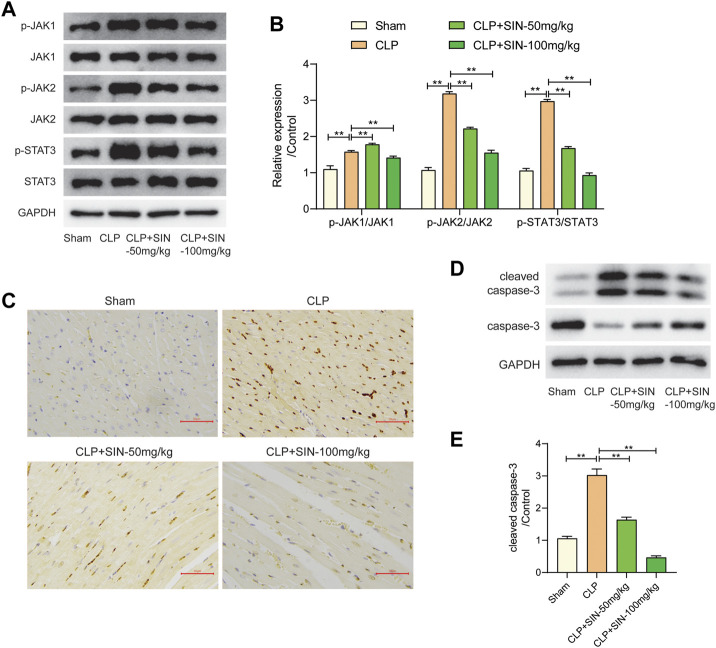
The effects of SIN on the JAK/STAT pathway and cardiomyocyte apoptosis. **(A,B)** The expression levels of JAK1, JAK2, STAT3, and phosphorylated JAK1, JAK2, and STAT3 were assessed via Western blot. **(C)** The apoptosis level of cardiomyocytes was detected by TUNEL staining. **(D,E)** The protein expression of caspase-3 and cleaved-caspase-3 were evaluated. n = 6. *p < 0.05, **p < 0.01.

The JAK/STAT axis was associated with the progress of MI, and the biological processes involving JAK/STAT axis were found to be related to the protection of SIN from sepsis-induced MI. Here, we evaluated the impact of SIN on the phosphorylation levels of JAK1, JAK2, and STAT3, along with the apoptosis of myocardial tissues. The findings revealed a notable increase in the apoptotic level of cardiomyocytes and the expression of p-JAK1, p-JAK2 and p-STAT3 in the septic rats as compared with normal rats, whereas SIN treatment exhibited a dose-dependent effect in reducing cardiomyocyte apoptosis and phosphorylation of JAK1, JAK2 and STAT3 (Figures 7A,B). TUNEL assay performed on heart slices revealed an elevated number of TUNEL-positive nuclei in septic rats induced by CLP, whereas SIN demonstrated a dose-dependent effect in reducing the elevated number of TUNEL-positive nuclei in septic rats (Figure 7C). The ratio of cleaved-casp3/caspase-3 was significantly increased in the septic rats compared to the normal rats, but rats with sepsis that received SIN treatment showed a lower ratio of cleaved casp3/caspase-3 (Figures 7D,E). These data corroborated the apoptosis results detected by TUNEL assays. These finding suggested that SIN treatment might ameliorate activated cardiomyocyte apoptosis in rats with sepsis via the JAK/STAT signal pathway.

## 4 Discussion

Sepsis, a major contributor to infection-related death, poses a challenge for healthcare systems around the world. The key to treating sepsis in clinical settings revolves around the administration of antimicrobial medicines to combat underlying infection. In recent decades, natural compounds exhibiting antibacterial and anti-inflammatory properties have been increasing utilized for the prevention of human diseases during the past few decades (McBride et al., 2020). Natural alkaloid SIN, having anti-inflammatory, immunoregulatory (Liu et al., 2020), anti-angiogenic (Feng et al., 2019) and other diverse pharmacological effects, is obtained from Sinomenium acutum Rehder. Previous studies have confirmed that SIN improved lung injury in sepsis by mediating gut homeostasis (Wang et al., 2020), and its hydrochloride salt could protect against polymicrobial sepsis via autophagy (Jiang et al., 2015). However, the potential therapeutic effects of SIN on sepsis-induced MI remain unclear. The findings of the present study suggested that SIN was favorable to improve the cardiac dysfunction of rats with sepsis, which was specifically manifested in the aspects of reducing the mortality rate, improving the cardiac functions, as well as ameliorating the MI of septic rats. Accumulating evidence has demonstrated the multiple and diverse mechanisms underlying the diverse functions of SIN in different diseases. SIN has a significant inhibitory effect on Glioblastoma multiforme (GBM) in advanced gliomas (Zheng et al., 2021). It prevents acute lung injury in sepsis by regulating intestinal microbiota and restoring intestinal barrier through aryl hydrocarbon receptor/NRF2-dependent pathway (Song W. et al., 2021), and also regulates the rationality of neuroimmune interaction to exert analgesic effects (Lai et al., 2022). Nonetheless, the specific mechanism of the action of SIN on sepsis-induced MI remains poorly understood.

In this study, a total of 33 putative targets were obtained by merging the SIN-targets and sepsis-related targets, and there were 3 genes with a higher degree: TNF-α, IL-6, and IL-1β. Many studies have confirmed the regulation of SIN on the gene expression or secretion of these 3 cytokines in sepsis (Deng et al., 2022; Pei et al., 2022). The results of the current study also confirmed that SIN could induce increased secretion of TNF-α, IL-6, and IL-1β in CLP-induced sepsis. TNF-α is a vital pro-inflammatory cell cytokine that can trigger inflammatory cascades and cause multiple clinical symptoms in patients with sepsis, such as hypotension, disseminated intravascular coagulation, and organ failure. Recent studies suggested that TNF-α (−238 G/A) polymorphism was associated with the progression of sepsis (Georgescu et al., 2020). It has been considered as promising therapeutic target for treating sepsis and MI (Jin et al., 2013), and anti-TNF-α immunotherapy has been developed for treating sepsis (Qu et al., 2018). IL-6 is another pro-inflammatory cytokine that is critical in immune and inflammatory responses. High levels of IL-6 have been shown to be associated with an increased risk of severe sepsis and a higher mortality rate (Deng et al., 2022). Additionally, IL-6 (174G/C) polymorphism was proved to be associated with an increased susceptibility to sepsis (Hu et al., 2019). Uncoupling of IL-6 signaling and Microtubule-associated protein 1 light chain 3 (LC3)-associated phagocytosis was reported to cause immunoparalysis during sepsis (Akoumianaki et al., 2021). Genetic variants in IL-1β has been confirmed to be a risk factor for sepsis and MI (Varljen et al., 2020; Pan et al., 2021), and contribute to the clinical course of sepsis (Montoya-Ruiz et al., 2016). Therefore, it suggested that SIN might protect against sepsis-induced MI via targeting TNF-α, IL-6, and IL-1β.

Multiple pathways, including the IL-17 signal pathway, the NOD-like receptor signaling pathway, and the TNF signaling pathway, were identified to be prospective targets of SIN against sepsis. IL-17 is a pro-inflammatory cytokine that could activate Interleukin 16 (IL-16) production (Yao et al., 1995). It is crucial in the development of several malignancies, inflammatory and autoimmune diseases, and infectious diseases. It is of great pathophysiological significance in sepsis via IL-17-mediated response and signal transduction (Ge et al., 2020). In sepsis, recent investigations suggested that IL-17 may function as a biomarker and a therapeutic target (Bosmann and Ward, 2012; Ahmed Ali et al., 2018). In this study, there were nine targets of SIN involved in the IL-17 signal pathway, including JNK1, NF-κB, CXCL8, IL-6, TNF-α, cyclooxygenase-2 (COX2), IL-1β, IFN-γ, and Matrix metalloproteinase-9 (MMP9). According to the results of this study, SIN administration reduced the elevated secretion of inflammatory cytokines, including CXCL8, IL-6, TNF-α, COX2, IL-1β, and IFN-γ in the CLP model, and reversed the increased phosphorylation levels of JNK1 and NF-κB induced by CLP. It has been reported that SIN can reduce the phosphorylation levels of JNK1 and NF-κB in macrophages, thus slowing down the inflammatory response caused by Lipopolysaccharides (LSP)-induced sepsis (Teng et al., 2012). A series of compounds were revealed to be effective in improving the LSP-induced sepsis via activation or deactivation of JNK1 and NF-κB (Hsu et al., 2013; Rocca et al., 2021). Meanwhile, JNK1 and NF-κB are closely related to cardiac pathologies (Xia et al., 2016; Singh et al., 2020). As a result, it can be inferred that SIN regulated the inflammatory cytokines via targeting JNK1 and NF-κB in the IL-17 signal pathway, which could eventually contribute to the its protective role in sepsis-induced MI. This finding may be helpful in determining new therapy directions for sepsis-induced MI.

GO analysis demonstrated that the candidate targets of SIN against sepsis were mainly associated with biological processes of cytokines, inflammation, and protein phosphorylation. Notably, our findings indicate a robust engagement of the Janus Kinase-Signal Transducers and Activators of Transcription (JAK-STAT) axis of the candidate targets of SIN against sepsis-induced MI. Western blot analysis showed a significant regulation of the phosphorylation of JAK2 and STAT3 after SIN exposure in CLP rats. JAKs-STATs signal pathways, known as the pivotal downstream signaling components of cytokine receptors, play a crucial role in mediating the biological effects of cytokines (Villarino et al., 2017). It contributes to organ damage and other dysfunctions in sepsis and offers novel therapeutic possibilities for sepsis (Cai et al., 2015; Clere-Jehl et al., 2020). Moreover, the JAK-STAT pathway is an integral part of myocardial response to various cardiac injuries and plays a prominent role in cardioprotective therapies (Barry et al., 2007). Cardiomyocyte apoptosis is robustly confirmed to be associated with the development of sepsis (Li et al., 2019), and regulated via the JAK-STAT pathway (Zhang et al., 2022). Therefore, we detected the cardiomyocyte apoptosis in each group in this study, and the findings demonstrated that the level of cardiomyocyte apoptosis in SIN groups was much lower than that in the CLP group. These findings suggested that SIN may prevent MI in sepsis by controlling cardiomyocyte apoptosis through the JAK-STAT pathway.

## 5 Conclusion

In conclusion, SIN improved the mortality rate and cardiac function of septic rats, and ameliorated sepsis-induced MI. Potential targets and pathways of SIN against sepsis were identified through network pharmacology analysis integrating molecular docking simulation. The proposition that SIN protects against sepsis-induced MI via targeting multiple proteins and regulating cytokine secretion and cardiomyocyte apoptosis was finally experimentally validated.

## Data Availability

The original contributions presented in the study are included in the article/Supplementary Material, further inquiries can be directed to the corresponding author.
