# Supplementary figures and images for: Network pharmacology-based approach to explore the underlying mechanism of sinomenine on sepsis-induced myocardial injury in rats
Source: Front Pharmacol. 2023 Jun 14;14:1138858. doi: 10.3389/fphar.2023.1138858 (PMC10303801; doi:10.3389/fphar.2023.1138858)

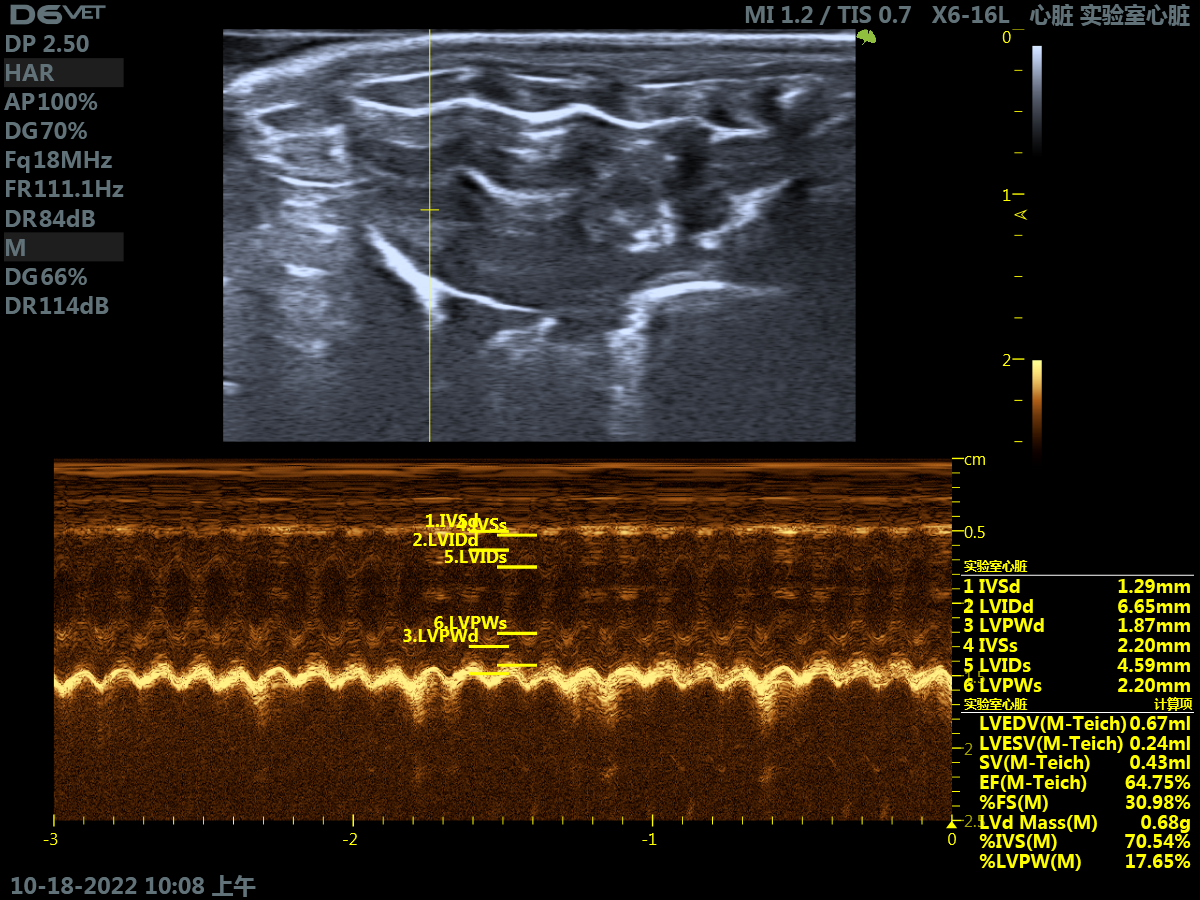

Supplement: Supplementary file 3 [file DataSheet1.ZIP › Raw Data/Echocardiography/CLP+SIN-100mgkg.png]

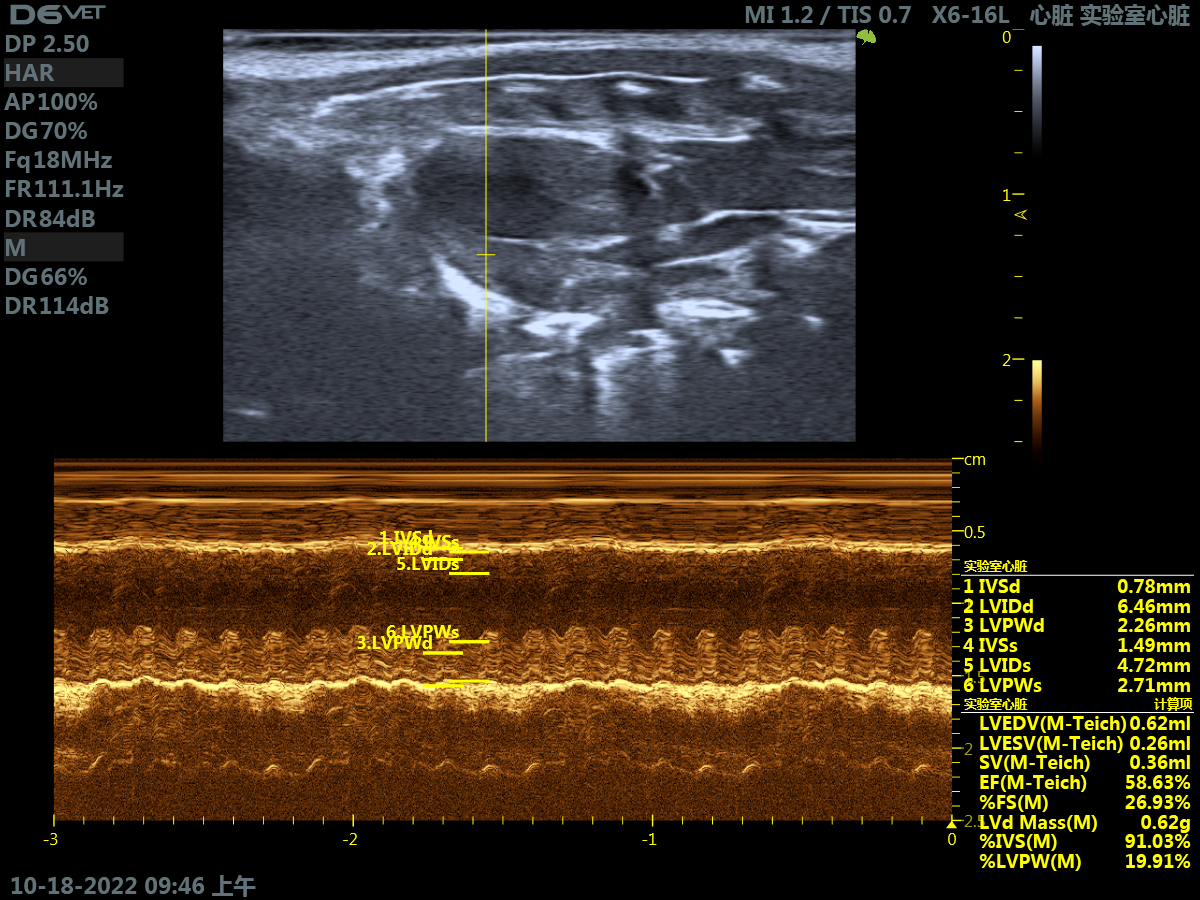

Supplement: Supplementary file 3 [file DataSheet1.ZIP › Raw Data/Echocardiography/CLP+SIN-50mgkg.png]

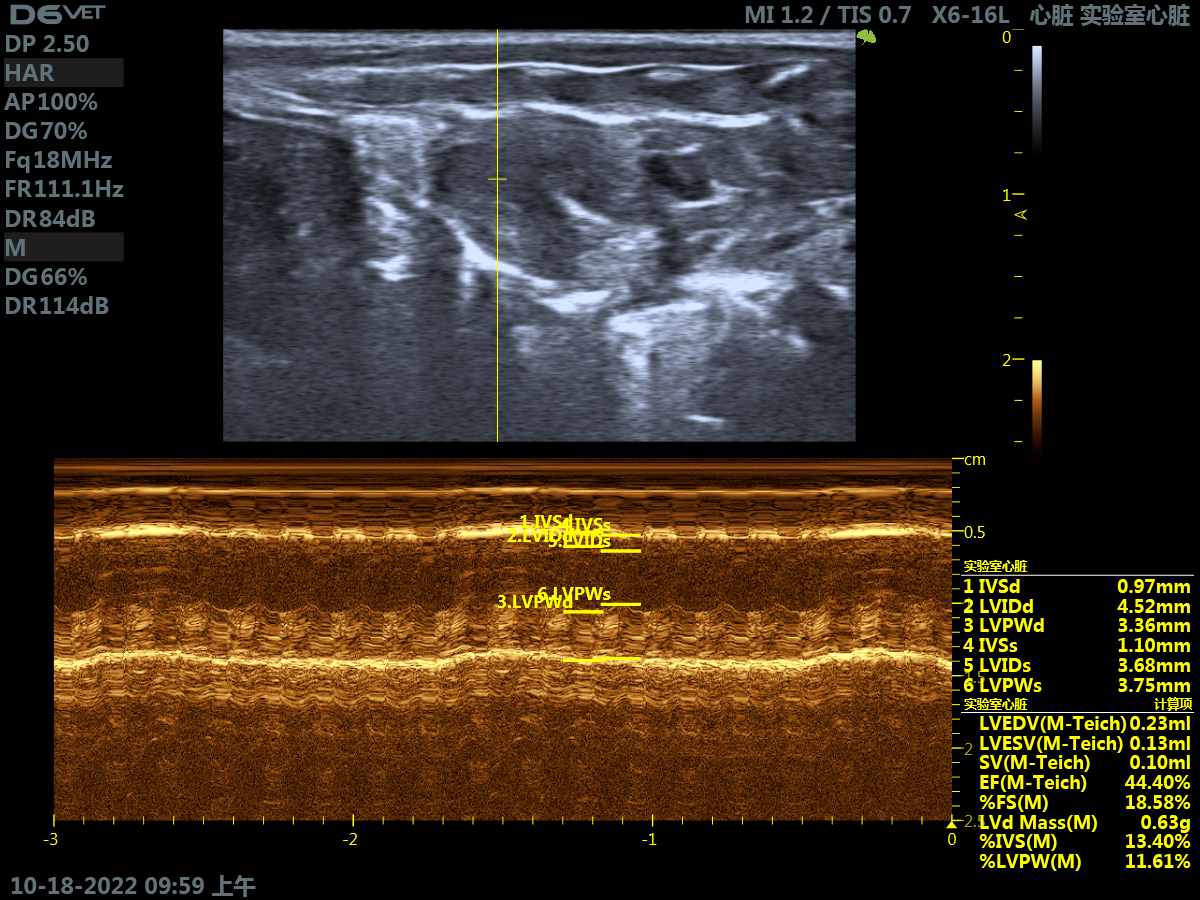

Supplement: Supplementary file 3 [file DataSheet1.ZIP › Raw Data/Echocardiography/CLP.png]

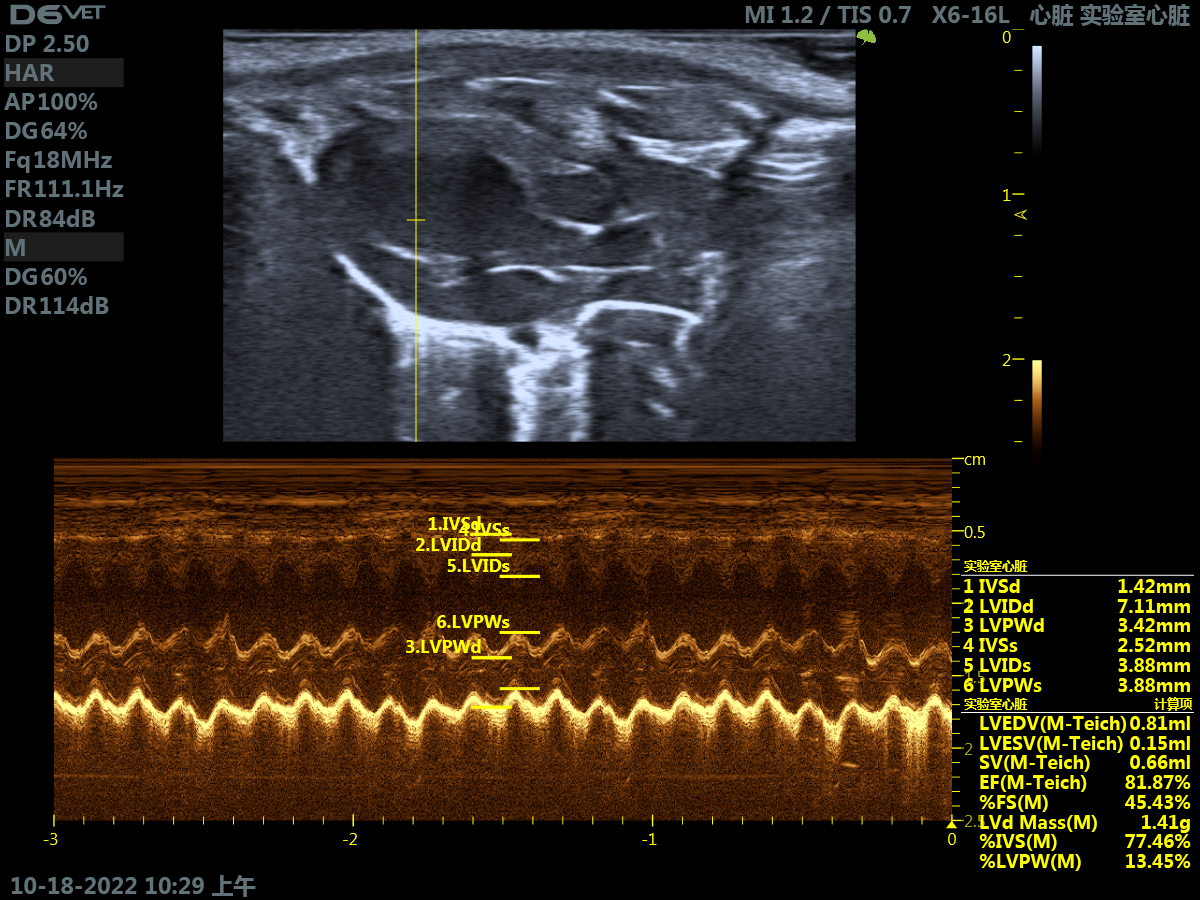

Supplement: Supplementary file 3 [file DataSheet1.ZIP › Raw Data/Echocardiography/Sham.png]

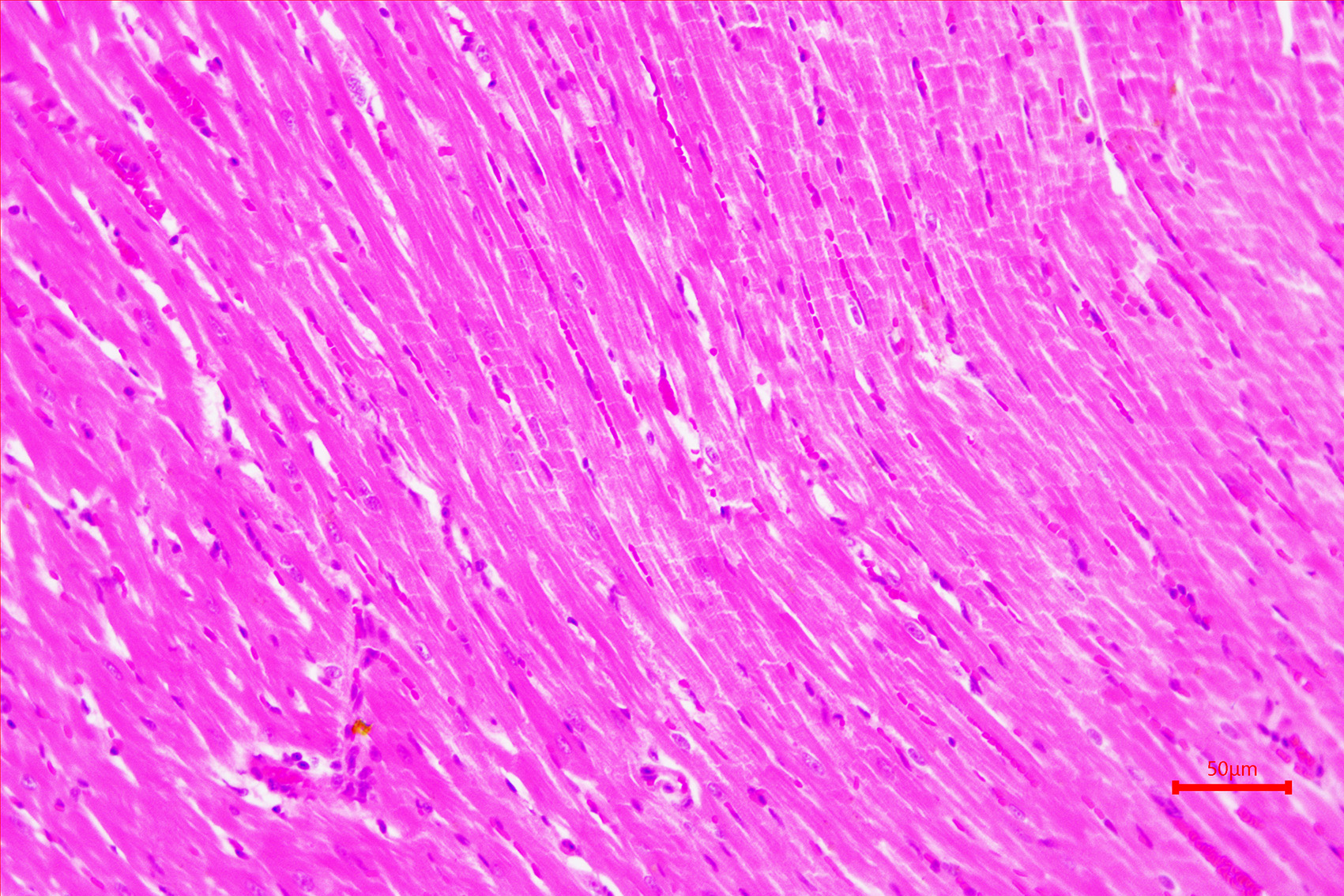

Supplement: Supplementary file 3 [file DataSheet1.ZIP › Raw Data/HE staining/CLP+SIN-100mgkg-1.jpg]

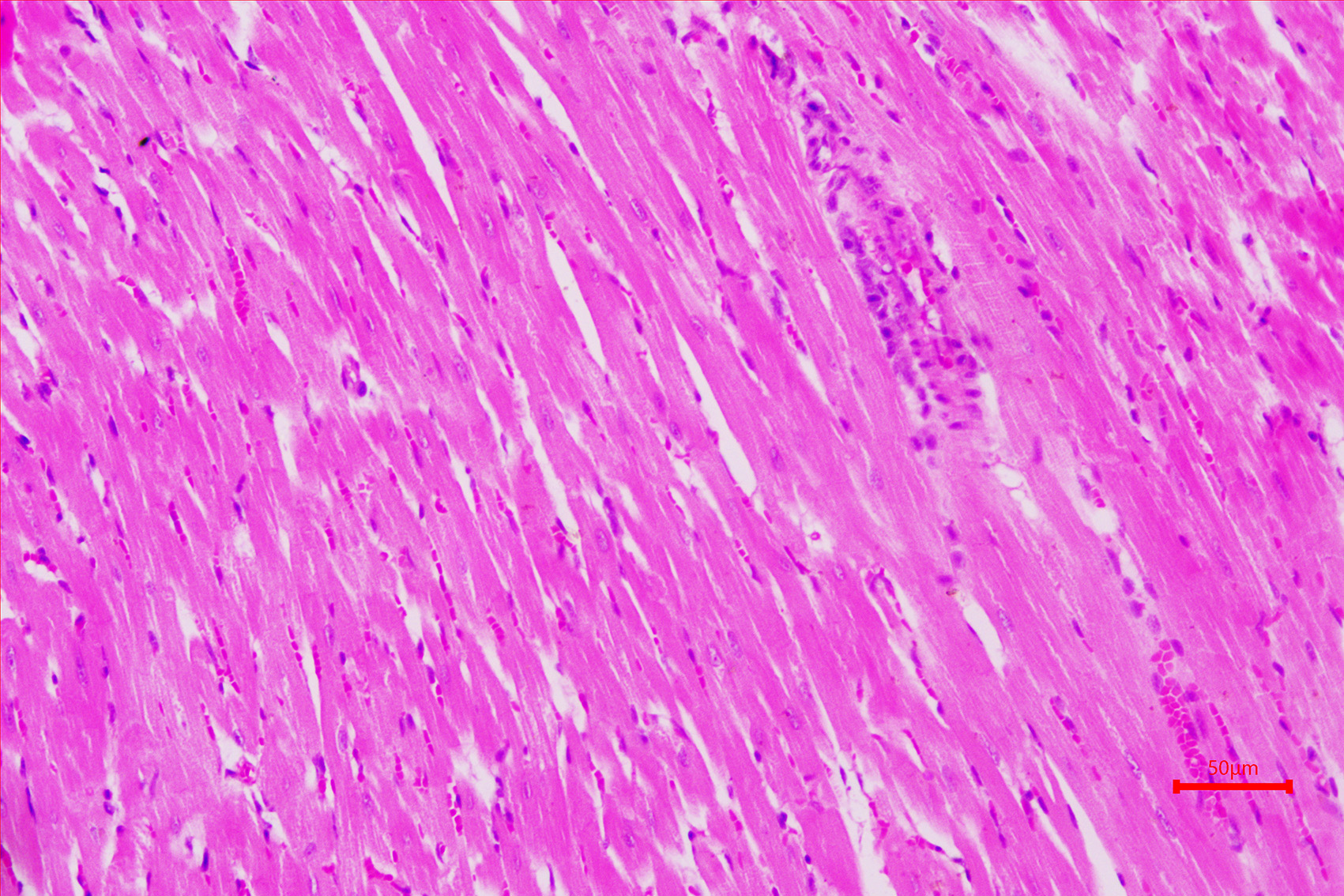

Supplement: Supplementary file 3 [file DataSheet1.ZIP › Raw Data/HE staining/CLP+SIN-50mgkg-1.jpg]

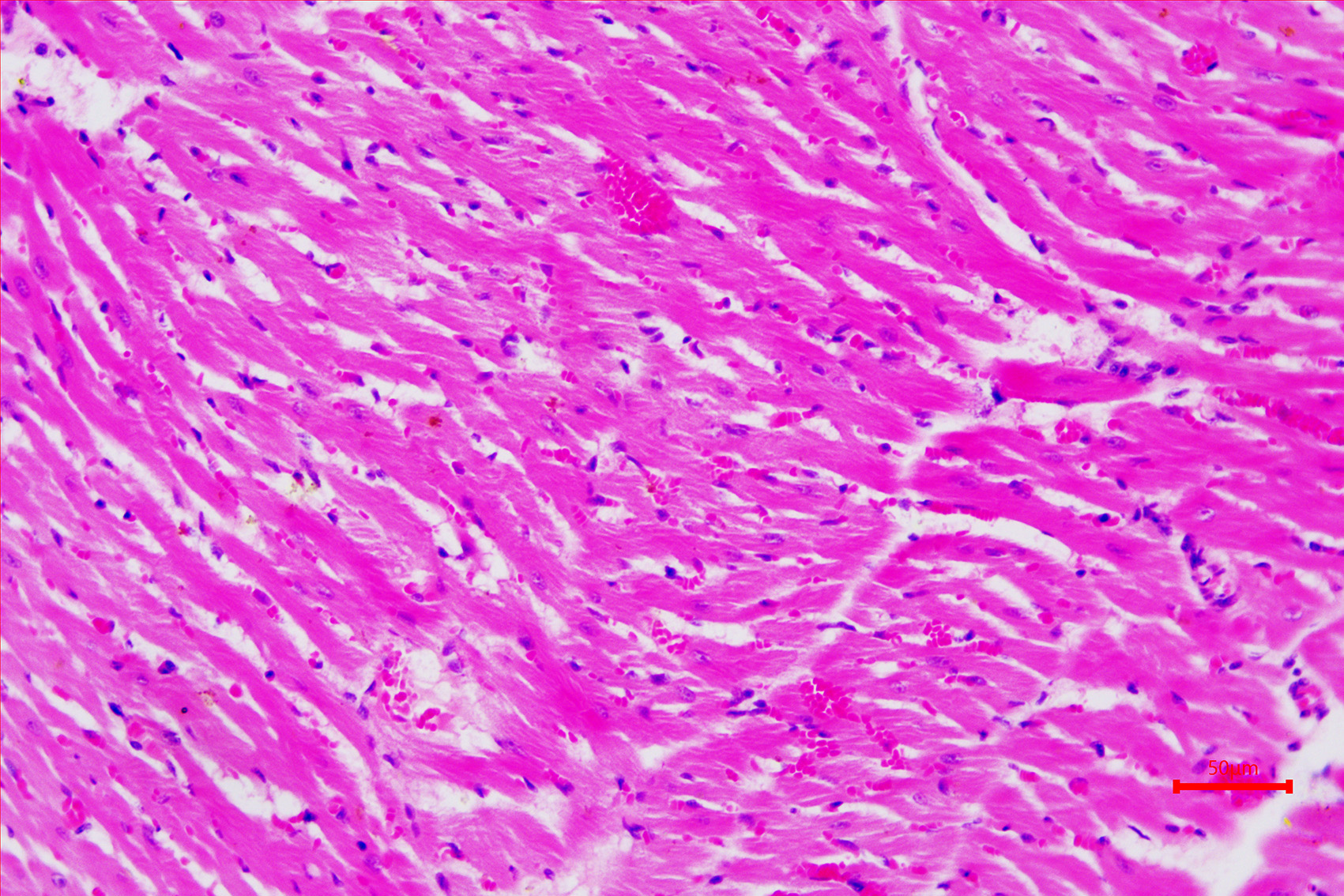

Supplement: Supplementary file 3 [file DataSheet1.ZIP › Raw Data/HE staining/CLP-1.jpg]

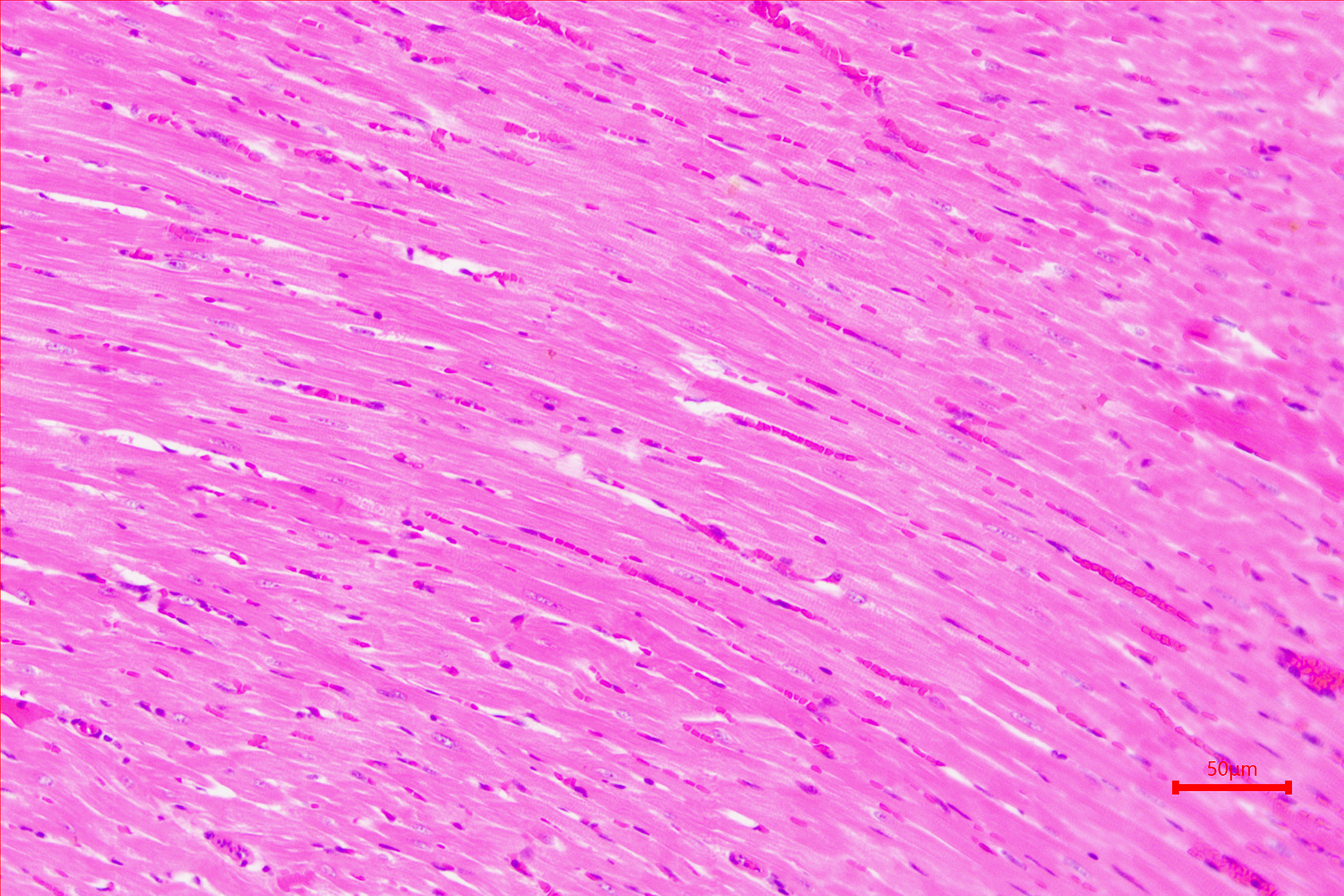

Supplement: Supplementary file 3 [file DataSheet1.ZIP › Raw Data/HE staining/Sham-1.jpg]

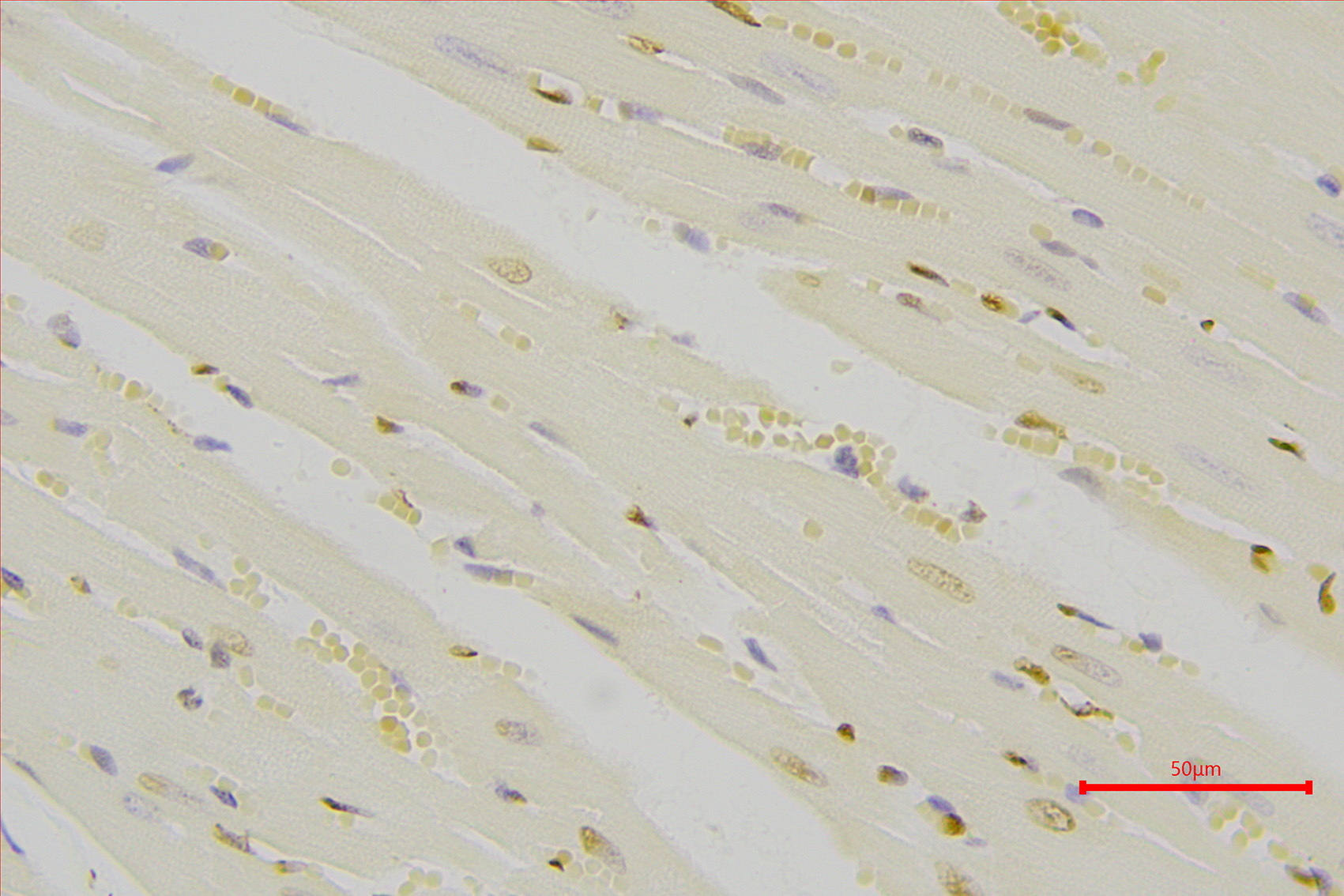

Supplement: Supplementary file 3 [file DataSheet1.ZIP › Raw Data/TUNEL staining/CLP+SIN-100mgkg.jpg]

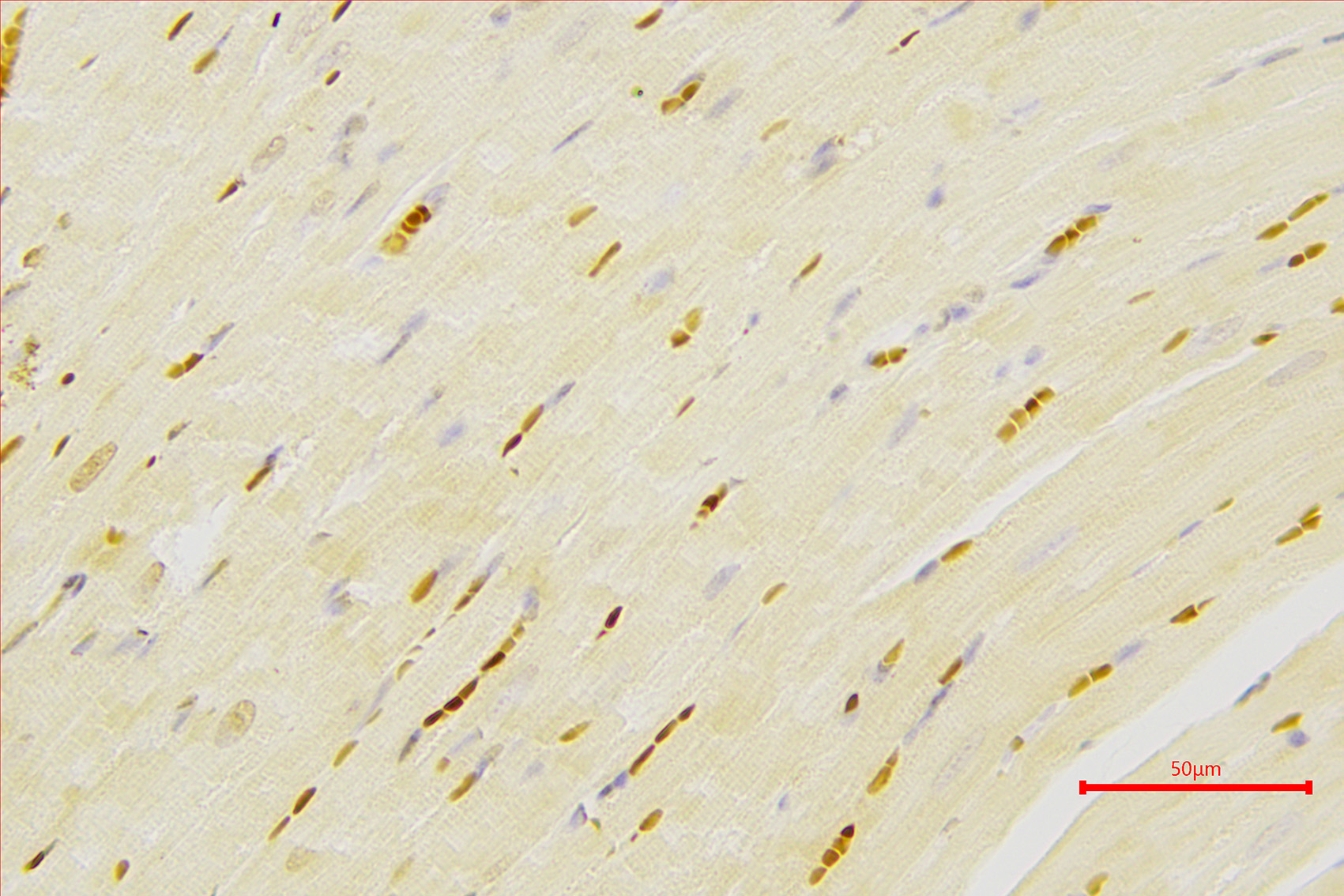

Supplement: Supplementary file 3 [file DataSheet1.ZIP › Raw Data/TUNEL staining/CLP+SIN-50mgkg.jpg]

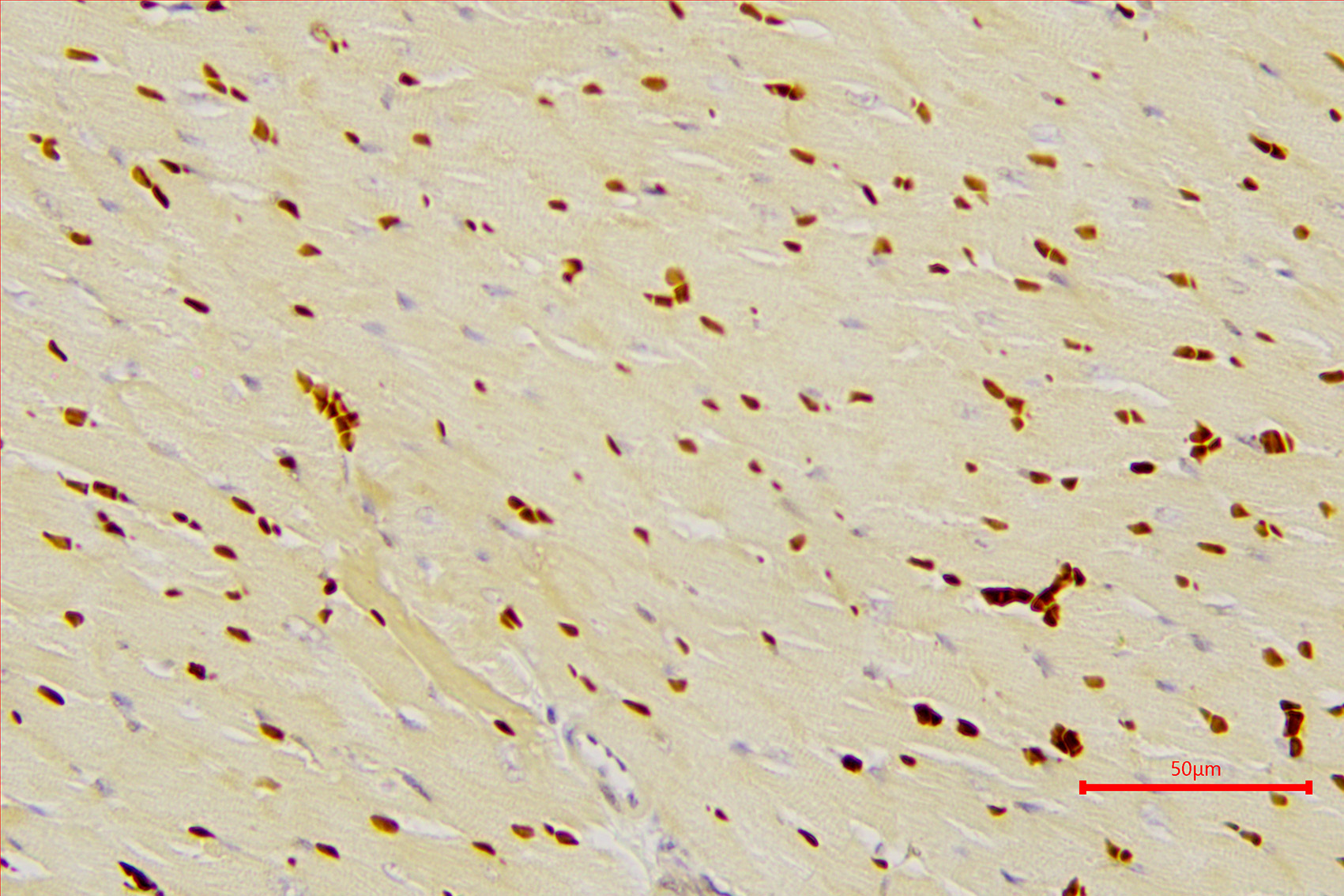

Supplement: Supplementary file 3 [file DataSheet1.ZIP › Raw Data/TUNEL staining/CLP.jpg]

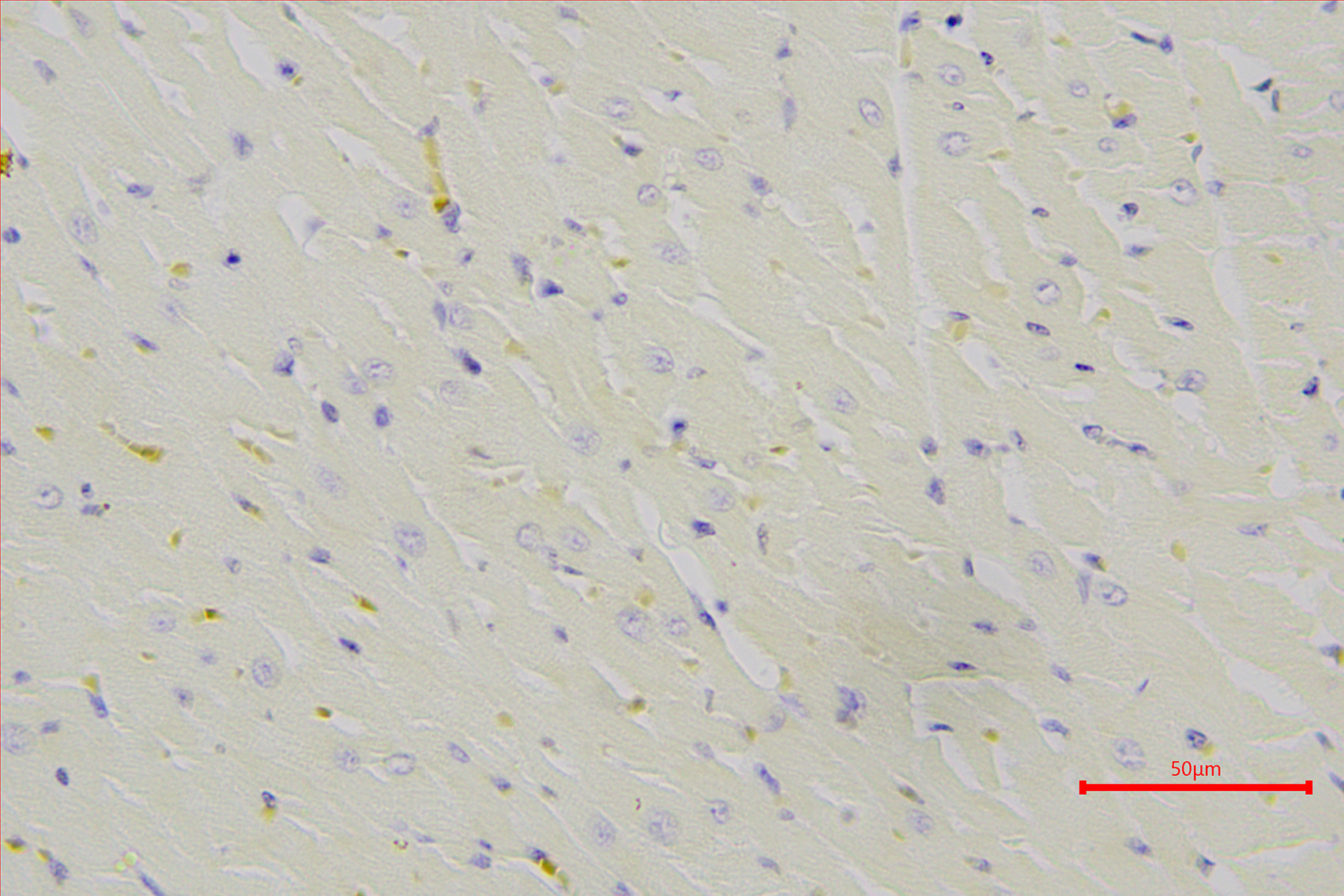

Supplement: Supplementary file 3 [file DataSheet1.ZIP › Raw Data/TUNEL staining/Sham.jpg]

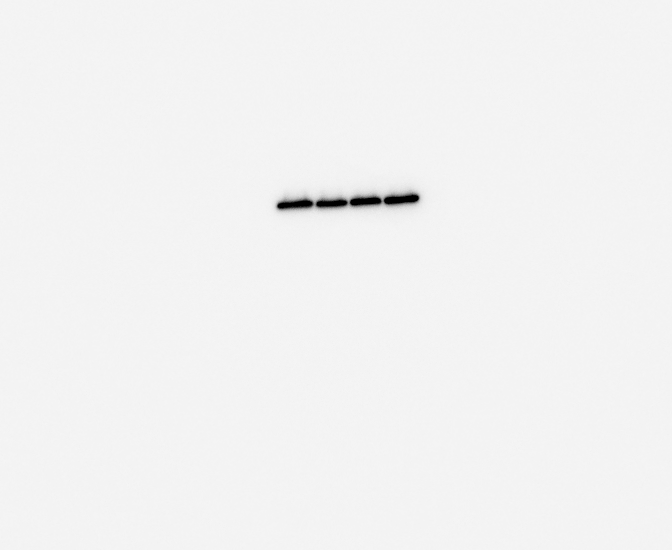

Supplement: Supplementary file 3 [file DataSheet1.ZIP › Raw Data/WB assay/Western Blotú¿Figure6ú⌐/GAPDH (1).tif]

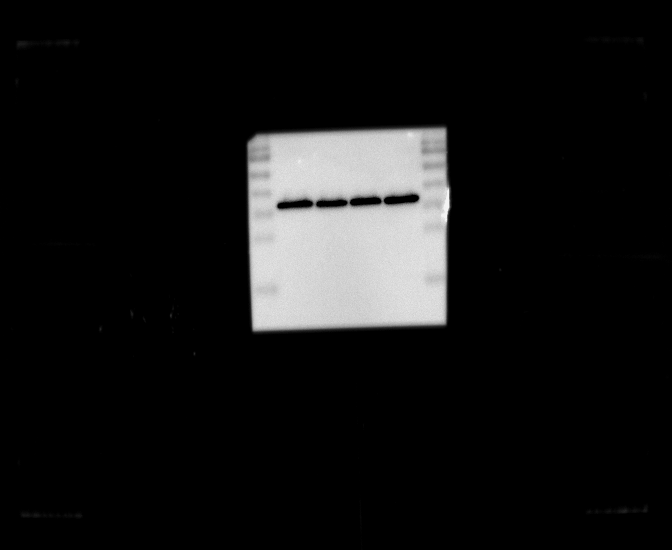

Supplement: Supplementary file 3 [file DataSheet1.ZIP › Raw Data/WB assay/Western Blotú¿Figure6ú⌐/GAPDH (2).tif]

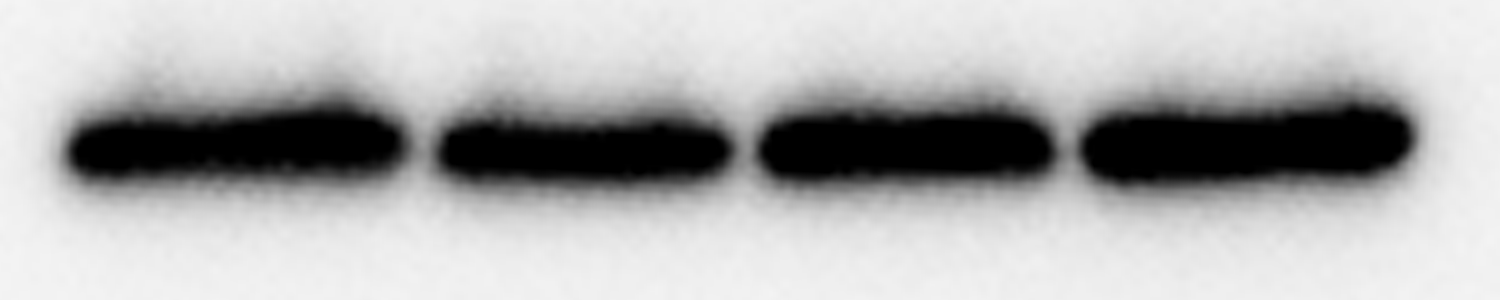

Supplement: Supplementary file 3 [file DataSheet1.ZIP › Raw Data/WB assay/Western Blotú¿Figure6ú⌐/GAPDH (3).tif]

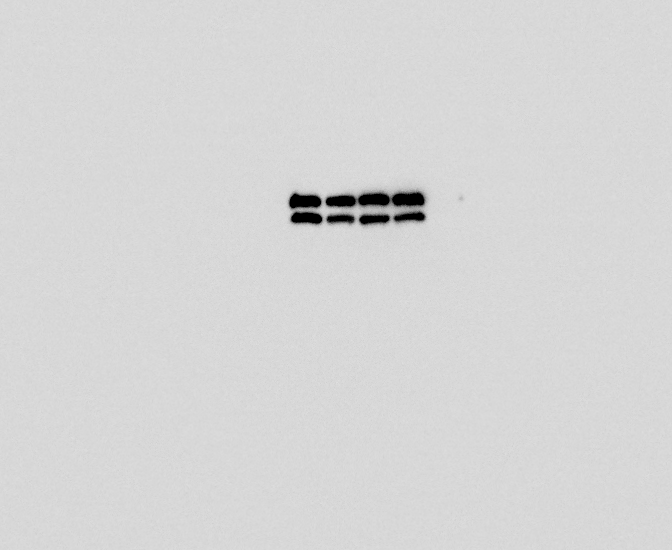

Supplement: Supplementary file 3 [file DataSheet1.ZIP › Raw Data/WB assay/Western Blotú¿Figure6ú⌐/JNK (1).tif]

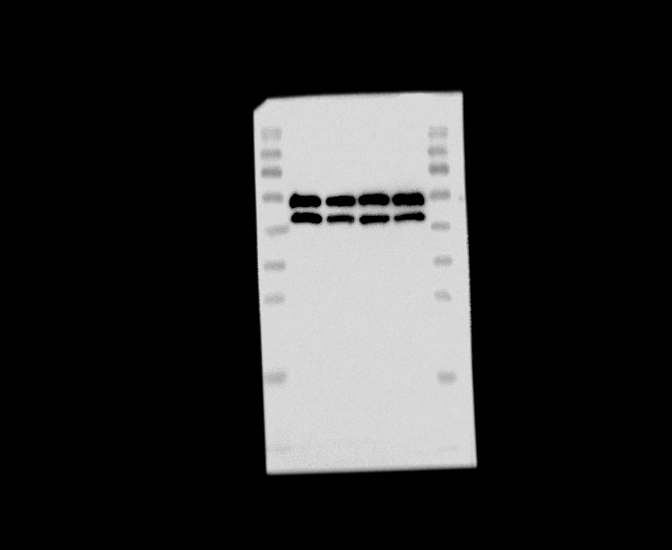

Supplement: Supplementary file 3 [file DataSheet1.ZIP › Raw Data/WB assay/Western Blotú¿Figure6ú⌐/JNK (2).tif]

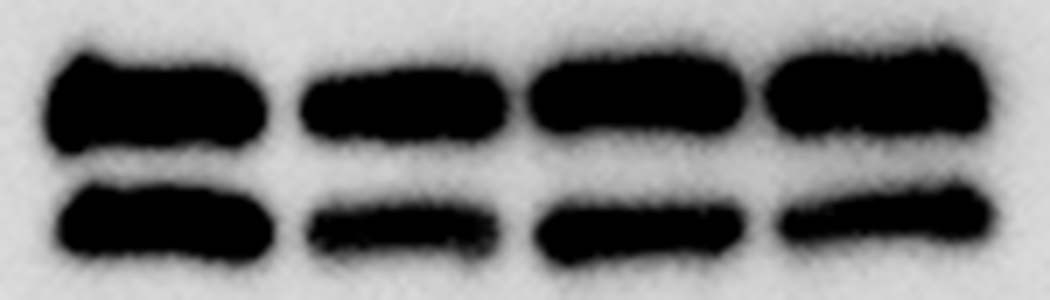

Supplement: Supplementary file 3 [file DataSheet1.ZIP › Raw Data/WB assay/Western Blotú¿Figure6ú⌐/JNK (3).tif]

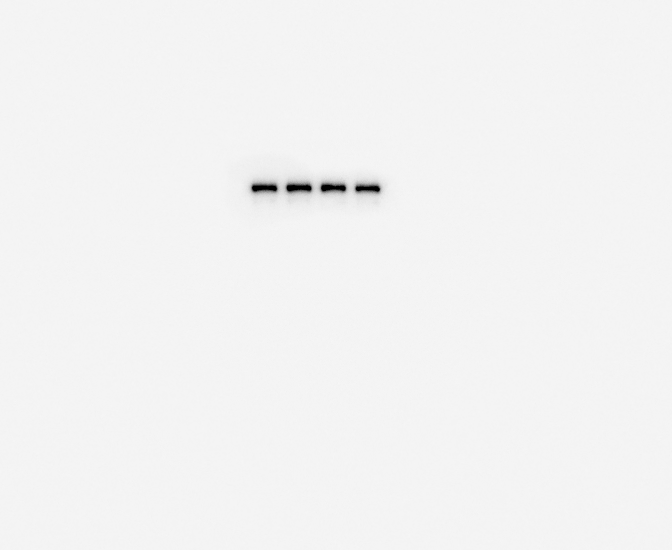

Supplement: Supplementary file 3 [file DataSheet1.ZIP › Raw Data/WB assay/Western Blotú¿Figure6ú⌐/NF-KB (1).tif]

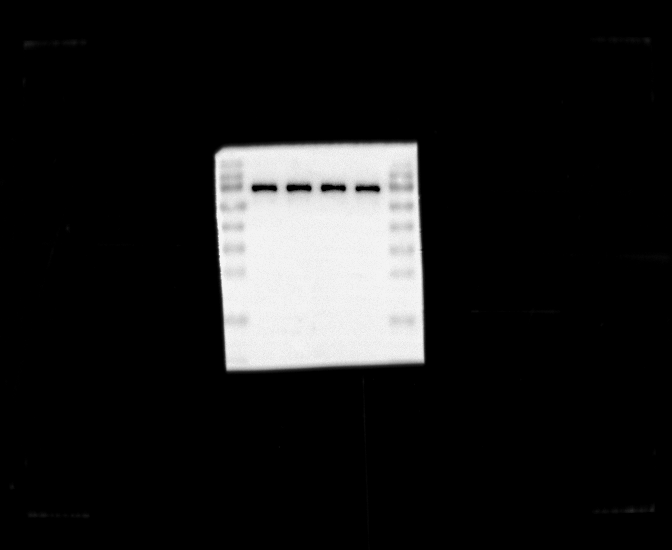

Supplement: Supplementary file 3 [file DataSheet1.ZIP › Raw Data/WB assay/Western Blotú¿Figure6ú⌐/NF-KB (2).tif]

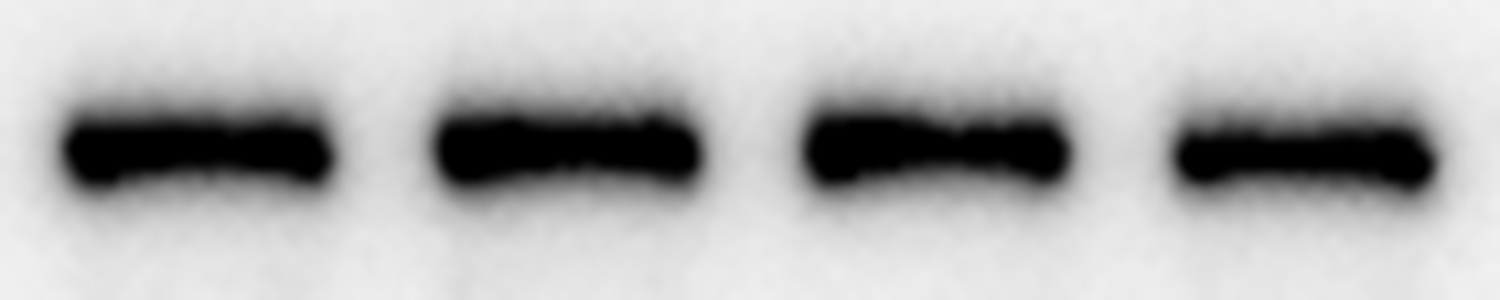

Supplement: Supplementary file 3 [file DataSheet1.ZIP › Raw Data/WB assay/Western Blotú¿Figure6ú⌐/NF-KB (3).tif]

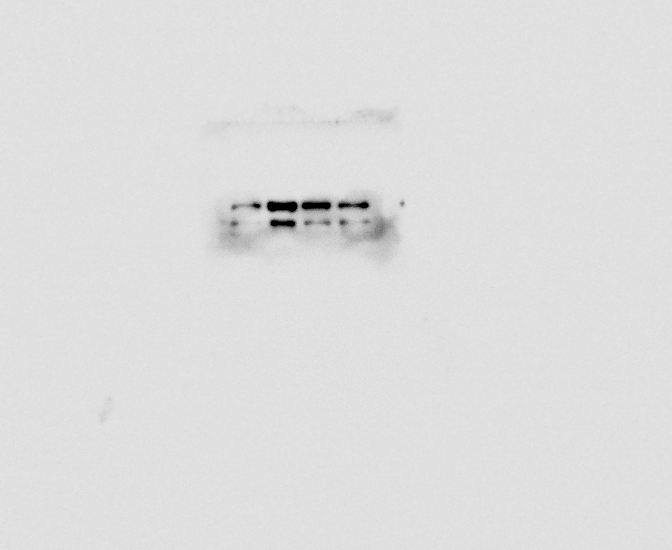

Supplement: Supplementary file 3 [file DataSheet1.ZIP › Raw Data/WB assay/Western Blotú¿Figure6ú⌐/P-JNK (1).tif]

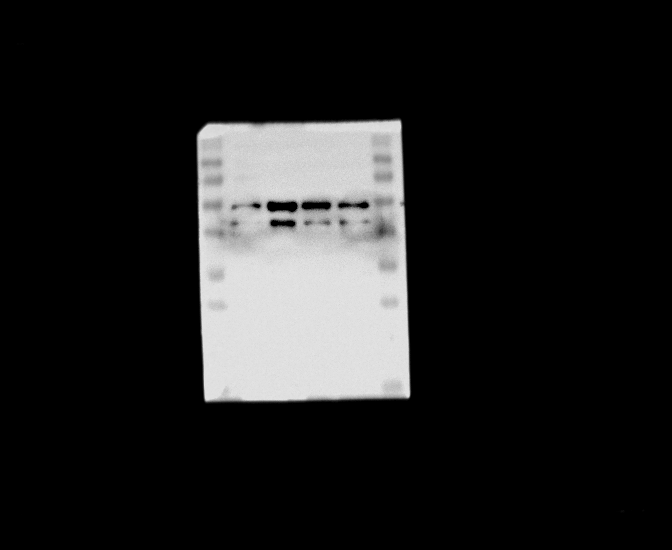

Supplement: Supplementary file 3 [file DataSheet1.ZIP › Raw Data/WB assay/Western Blotú¿Figure6ú⌐/P-JNK (2).tif]

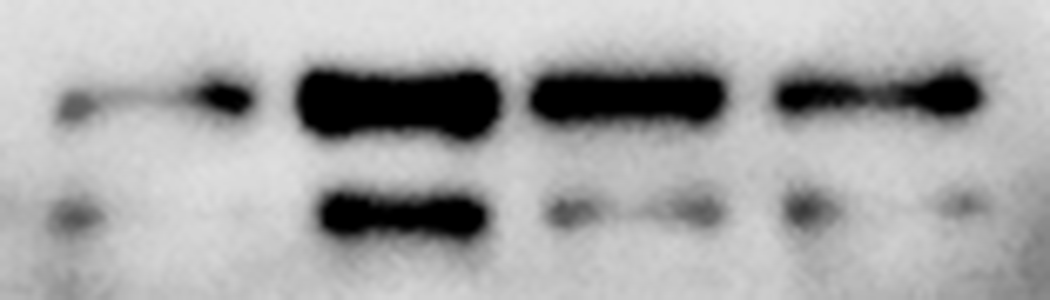

Supplement: Supplementary file 3 [file DataSheet1.ZIP › Raw Data/WB assay/Western Blotú¿Figure6ú⌐/P-JNK (3).tif]

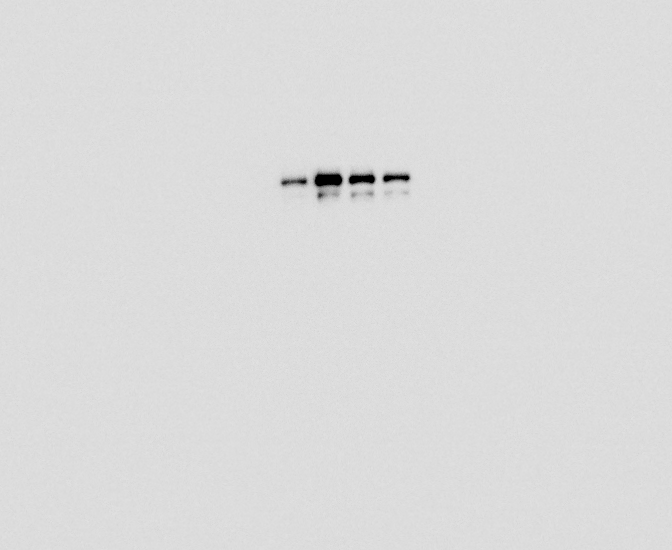

Supplement: Supplementary file 3 [file DataSheet1.ZIP › Raw Data/WB assay/Western Blotú¿Figure6ú⌐/p-NF-KB (1).tif]

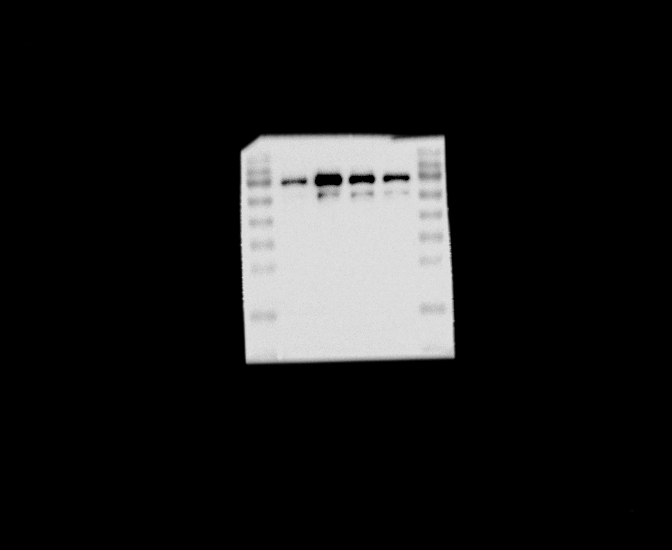

Supplement: Supplementary file 3 [file DataSheet1.ZIP › Raw Data/WB assay/Western Blotú¿Figure6ú⌐/p-NF-KB (2).tif]

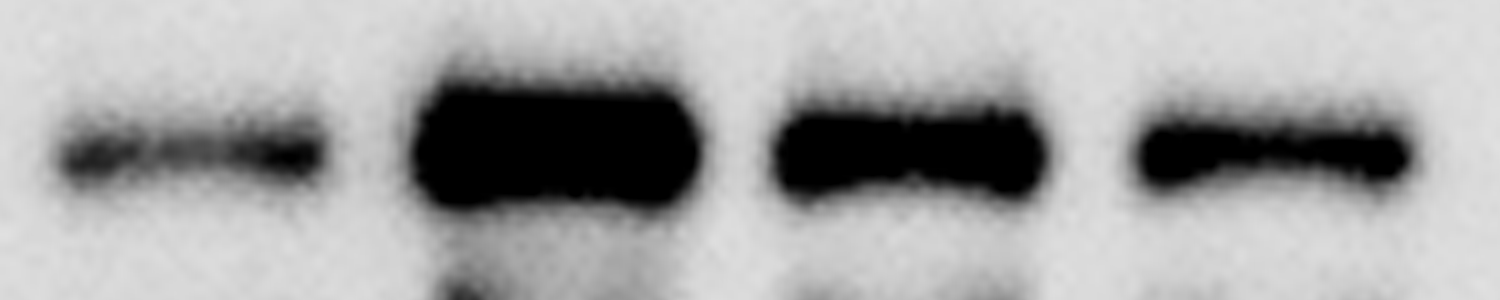

Supplement: Supplementary file 3 [file DataSheet1.ZIP › Raw Data/WB assay/Western Blotú¿Figure6ú⌐/p-NF-KB (3).tif]

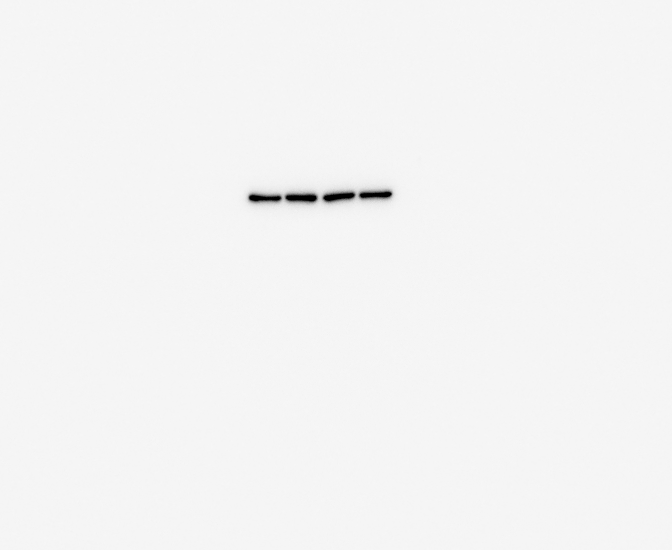

Supplement: Supplementary file 3 [file DataSheet1.ZIP › Raw Data/WB assay/Western Blotú¿Figure7ú⌐/Fig 7A/GAPDH (1).tif]

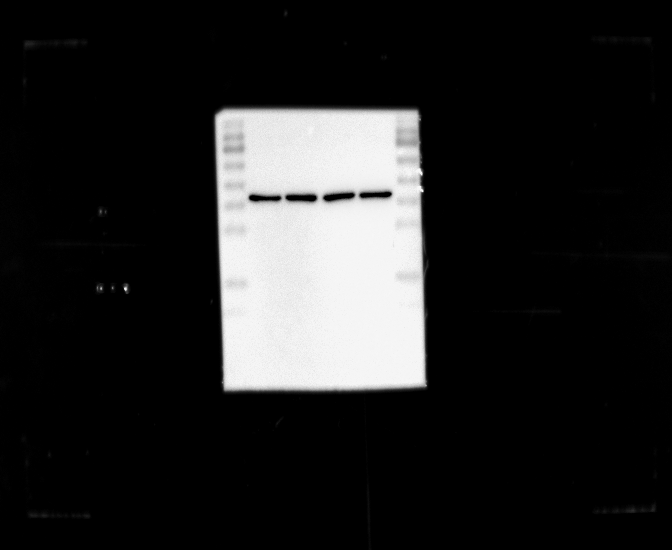

Supplement: Supplementary file 3 [file DataSheet1.ZIP › Raw Data/WB assay/Western Blotú¿Figure7ú⌐/Fig 7A/GAPDH (2).tif]

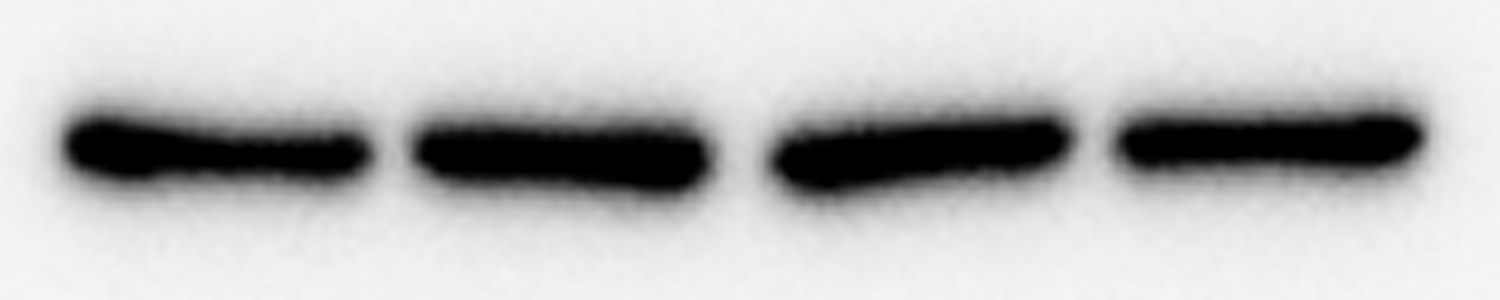

Supplement: Supplementary file 3 [file DataSheet1.ZIP › Raw Data/WB assay/Western Blotú¿Figure7ú⌐/Fig 7A/GAPDH (3).tif]

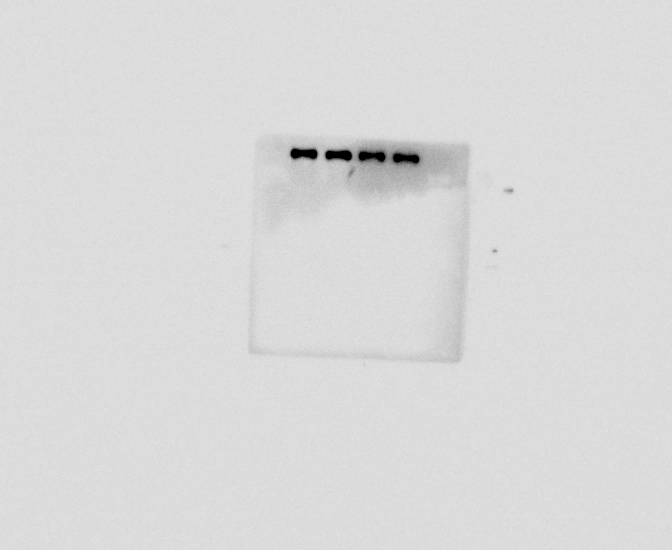

Supplement: Supplementary file 3 [file DataSheet1.ZIP › Raw Data/WB assay/Western Blotú¿Figure7ú⌐/Fig 7A/JAK1/JAK1 (1).tif]

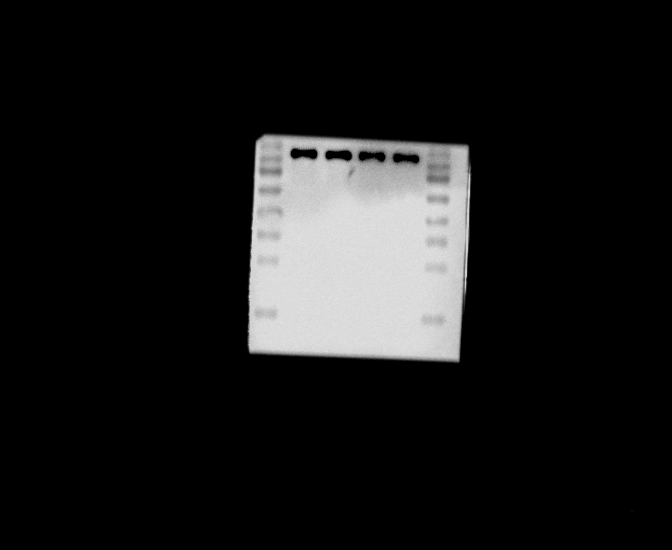

Supplement: Supplementary file 3 [file DataSheet1.ZIP › Raw Data/WB assay/Western Blotú¿Figure7ú⌐/Fig 7A/JAK1/JAK1 (2).tif]

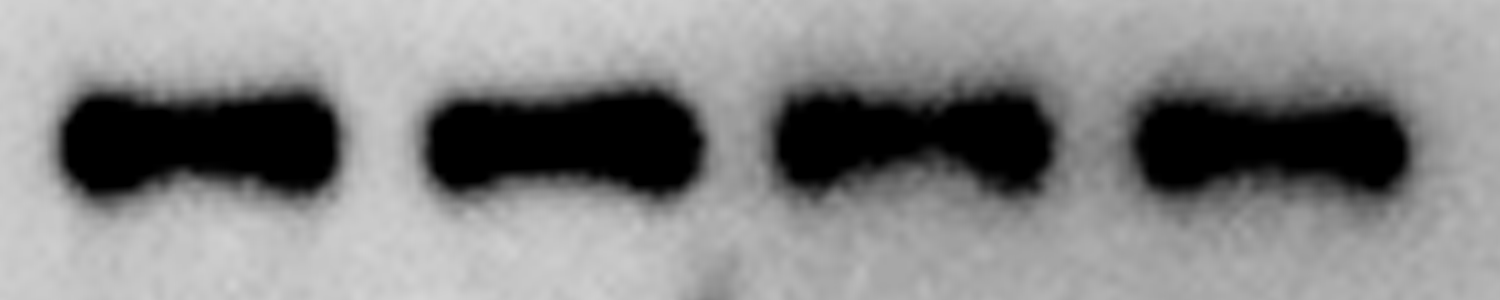

Supplement: Supplementary file 3 [file DataSheet1.ZIP › Raw Data/WB assay/Western Blotú¿Figure7ú⌐/Fig 7A/JAK1/JAK1 (3).tif]

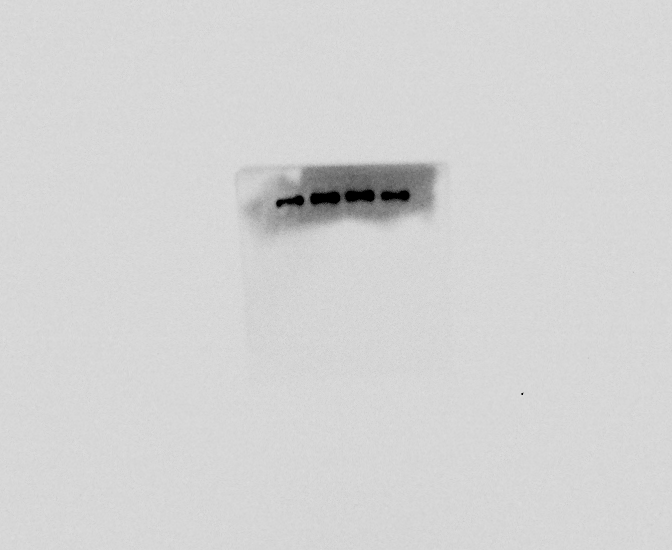

Supplement: Supplementary file 3 [file DataSheet1.ZIP › Raw Data/WB assay/Western Blotú¿Figure7ú⌐/Fig 7A/JAK1/p-JAK1 (1).tif]

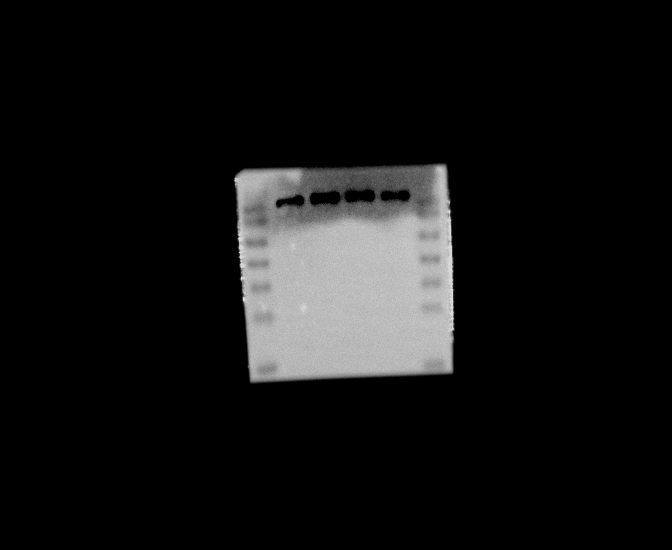

Supplement: Supplementary file 3 [file DataSheet1.ZIP › Raw Data/WB assay/Western Blotú¿Figure7ú⌐/Fig 7A/JAK1/p-JAK1 (2).tif]

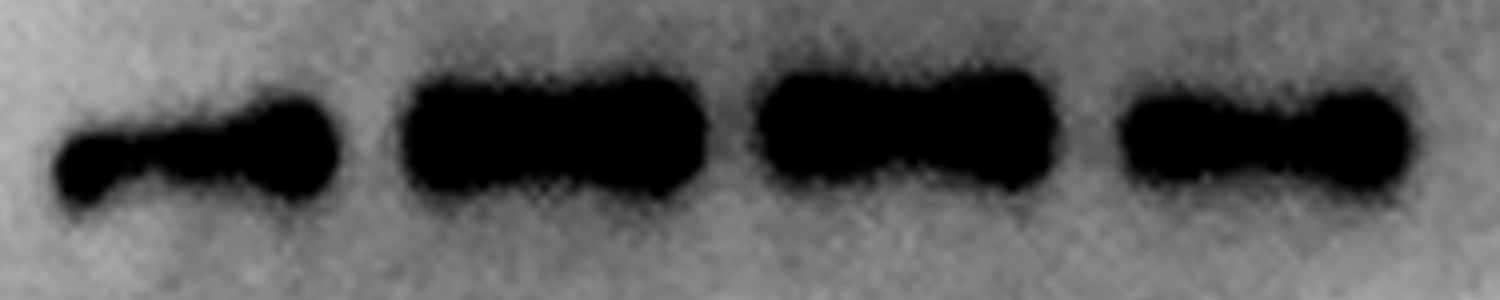

Supplement: Supplementary file 3 [file DataSheet1.ZIP › Raw Data/WB assay/Western Blotú¿Figure7ú⌐/Fig 7A/JAK1/p-JAK1 (3).tif]

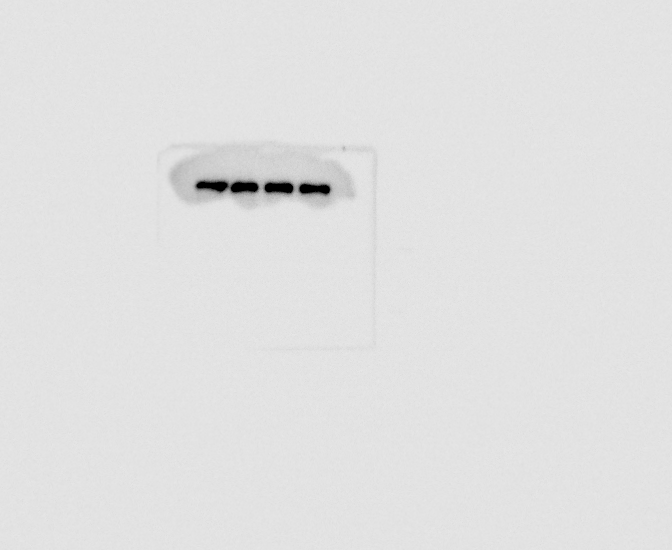

Supplement: Supplementary file 3 [file DataSheet1.ZIP › Raw Data/WB assay/Western Blotú¿Figure7ú⌐/Fig 7A/JAK2/JAK2 (1).tif]

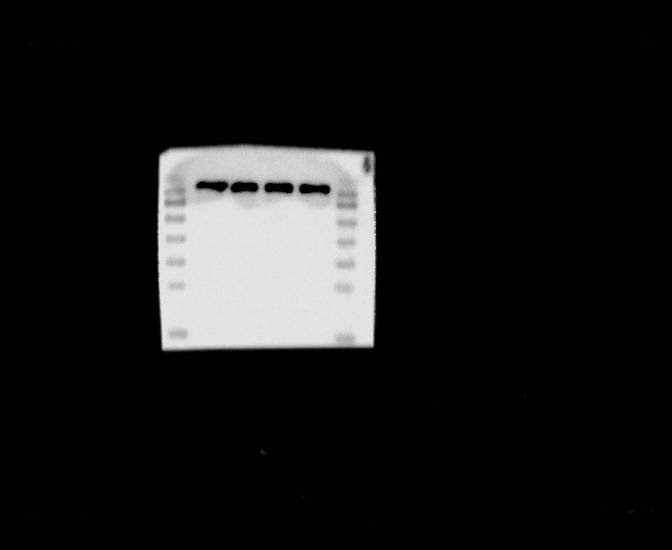

Supplement: Supplementary file 3 [file DataSheet1.ZIP › Raw Data/WB assay/Western Blotú¿Figure7ú⌐/Fig 7A/JAK2/JAK2 (2).tif]

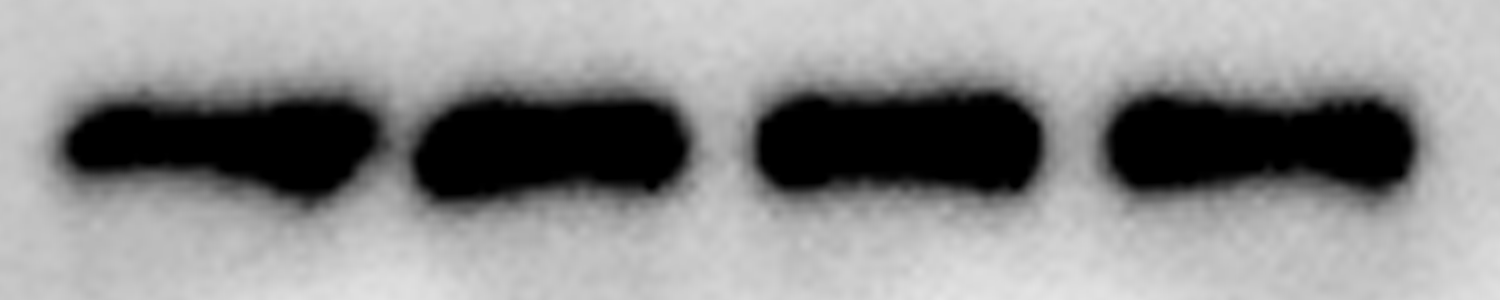

Supplement: Supplementary file 3 [file DataSheet1.ZIP › Raw Data/WB assay/Western Blotú¿Figure7ú⌐/Fig 7A/JAK2/JAK2 (3).tif]

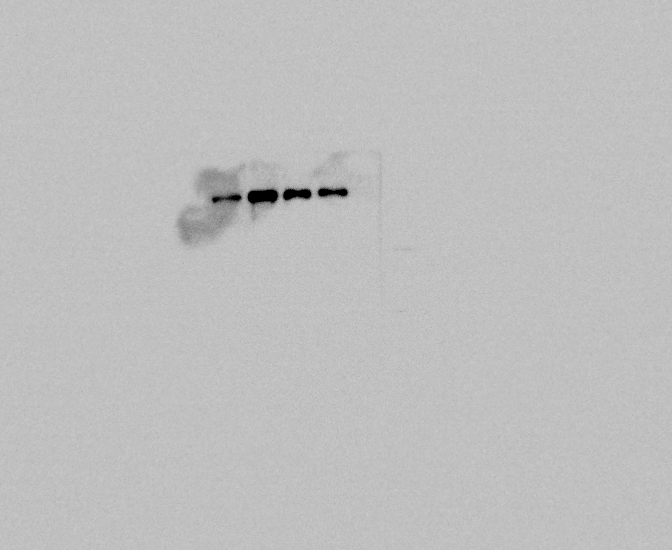

Supplement: Supplementary file 3 [file DataSheet1.ZIP › Raw Data/WB assay/Western Blotú¿Figure7ú⌐/Fig 7A/JAK2/p-JAK2 (1).tif]

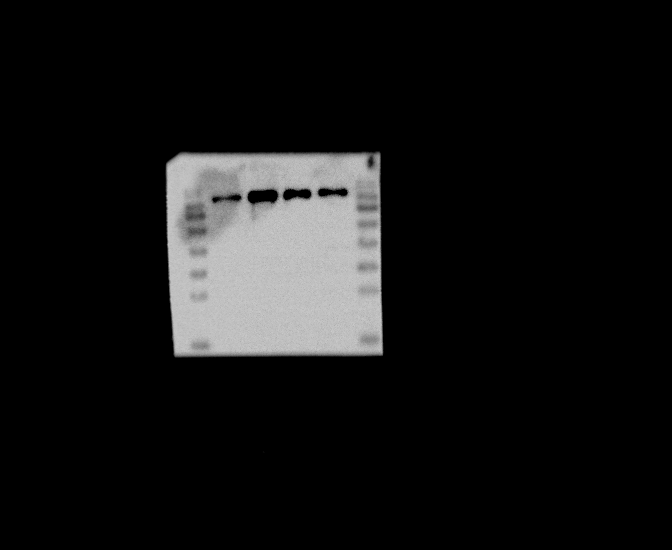

Supplement: Supplementary file 3 [file DataSheet1.ZIP › Raw Data/WB assay/Western Blotú¿Figure7ú⌐/Fig 7A/JAK2/p-JAK2 (2).tif]

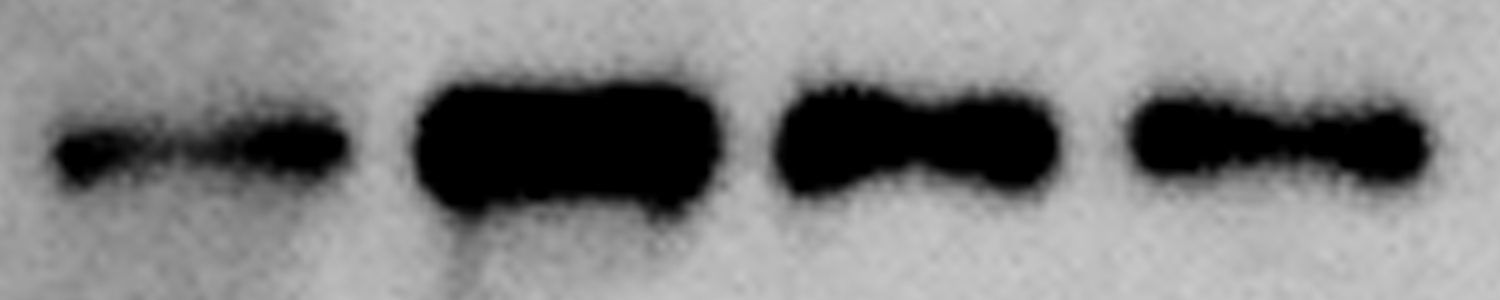

Supplement: Supplementary file 3 [file DataSheet1.ZIP › Raw Data/WB assay/Western Blotú¿Figure7ú⌐/Fig 7A/JAK2/p-JAK2 (3).tif]

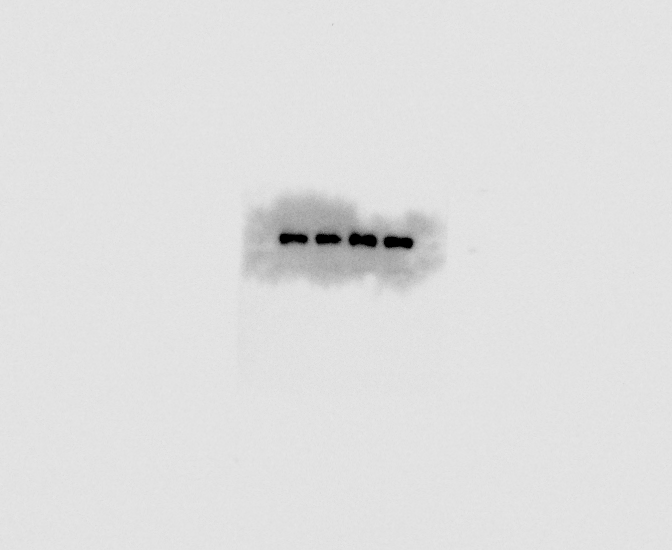

Supplement: Supplementary file 3 [file DataSheet1.ZIP › Raw Data/WB assay/Western Blotú¿Figure7ú⌐/Fig 7A/stat/STAT3 (1).tif]

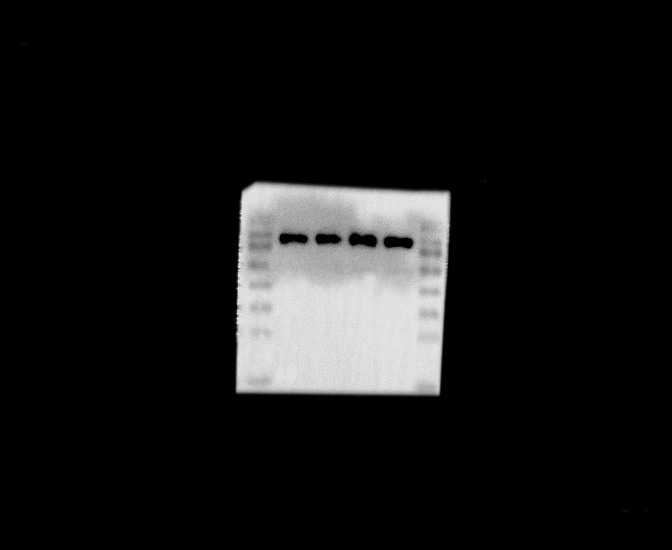

Supplement: Supplementary file 3 [file DataSheet1.ZIP › Raw Data/WB assay/Western Blotú¿Figure7ú⌐/Fig 7A/stat/STAT3 (2).tif]

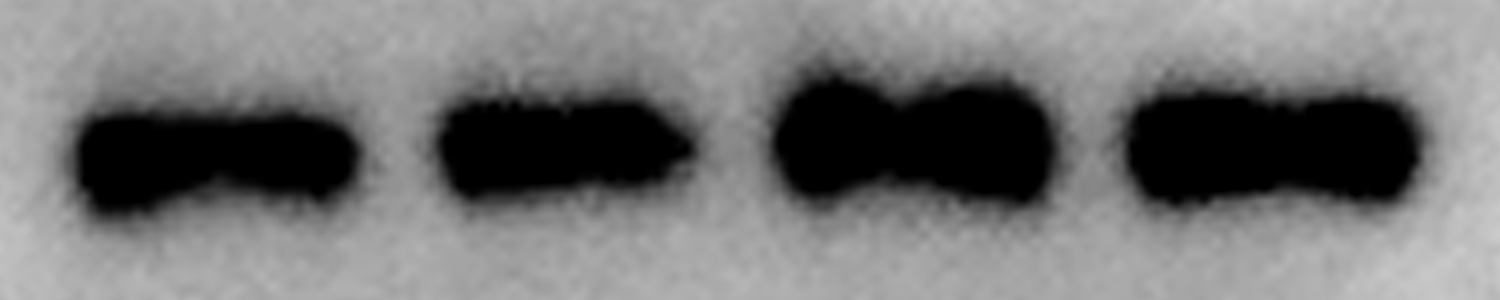

Supplement: Supplementary file 3 [file DataSheet1.ZIP › Raw Data/WB assay/Western Blotú¿Figure7ú⌐/Fig 7A/stat/STAT3 (3).tif]

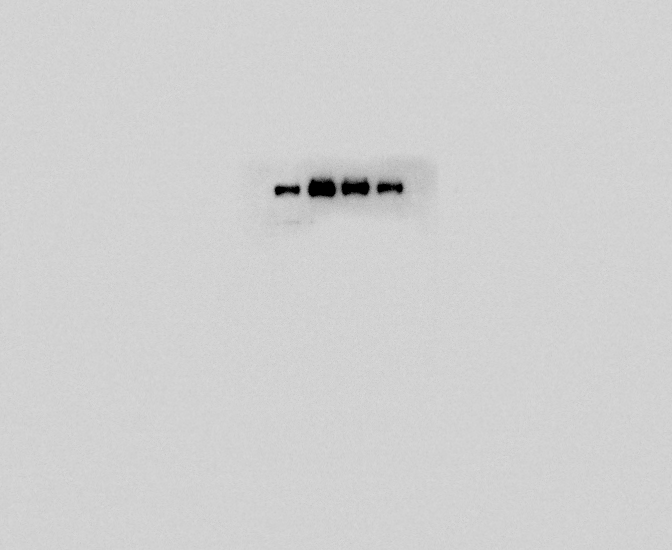

Supplement: Supplementary file 3 [file DataSheet1.ZIP › Raw Data/WB assay/Western Blotú¿Figure7ú⌐/Fig 7A/stat/p-STAT3 (1).tif]

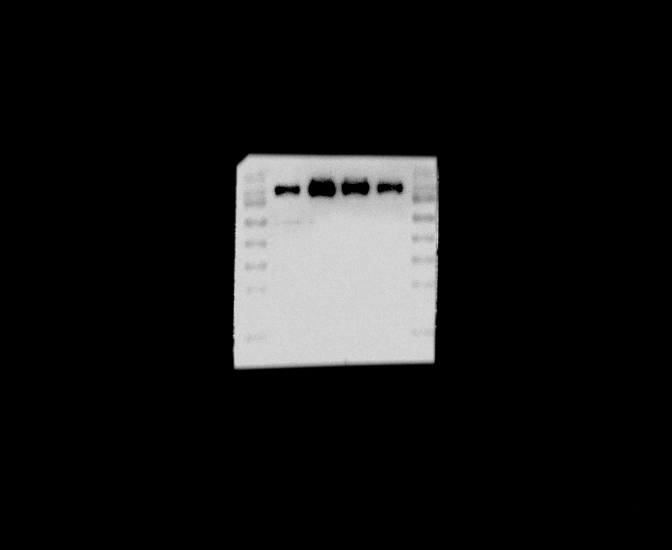

Supplement: Supplementary file 3 [file DataSheet1.ZIP › Raw Data/WB assay/Western Blotú¿Figure7ú⌐/Fig 7A/stat/p-STAT3 (2).tif]

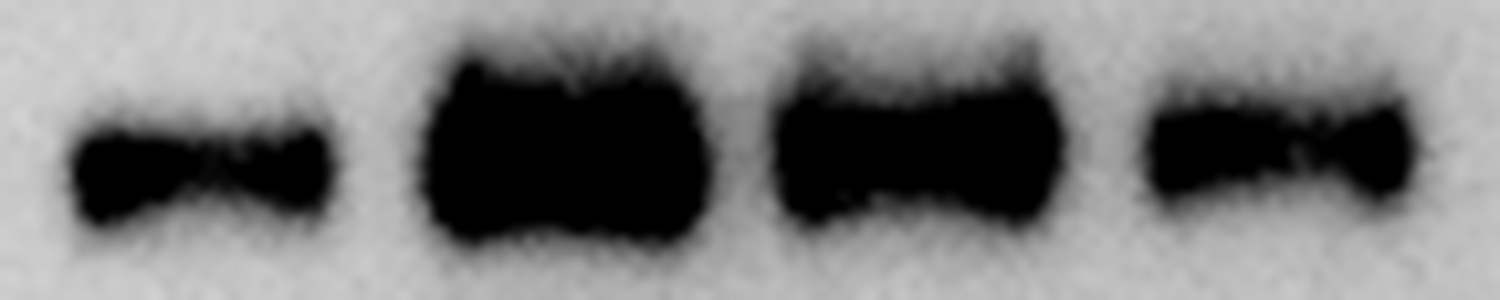

Supplement: Supplementary file 3 [file DataSheet1.ZIP › Raw Data/WB assay/Western Blotú¿Figure7ú⌐/Fig 7A/stat/p-STAT3 (3).tif]

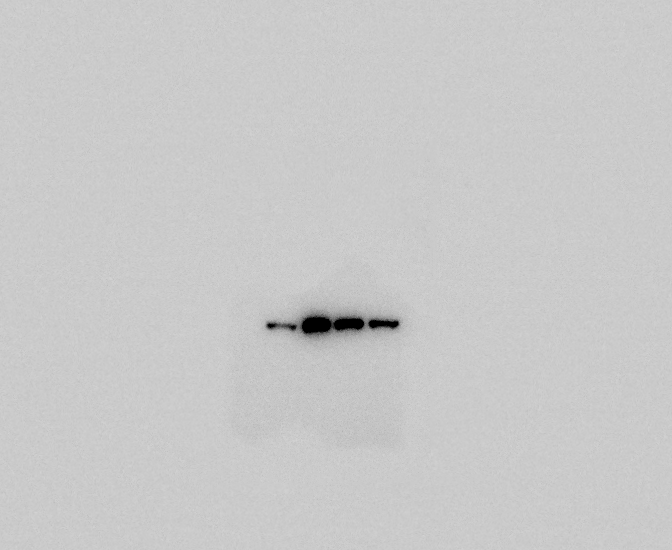

Supplement: Supplementary file 3 [file DataSheet1.ZIP › Raw Data/WB assay/Western Blotú¿Figure7ú⌐/Fig 7D/Cleaved_caspase3 (1).tif]

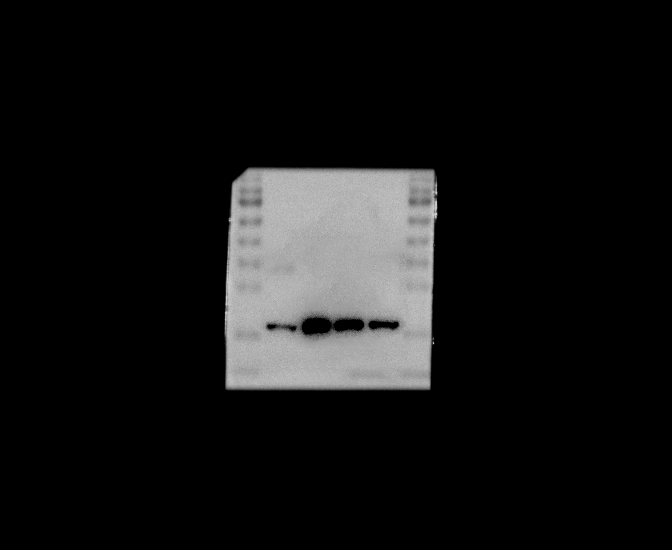

Supplement: Supplementary file 3 [file DataSheet1.ZIP › Raw Data/WB assay/Western Blotú¿Figure7ú⌐/Fig 7D/Cleaved_caspase3 (2).tif]

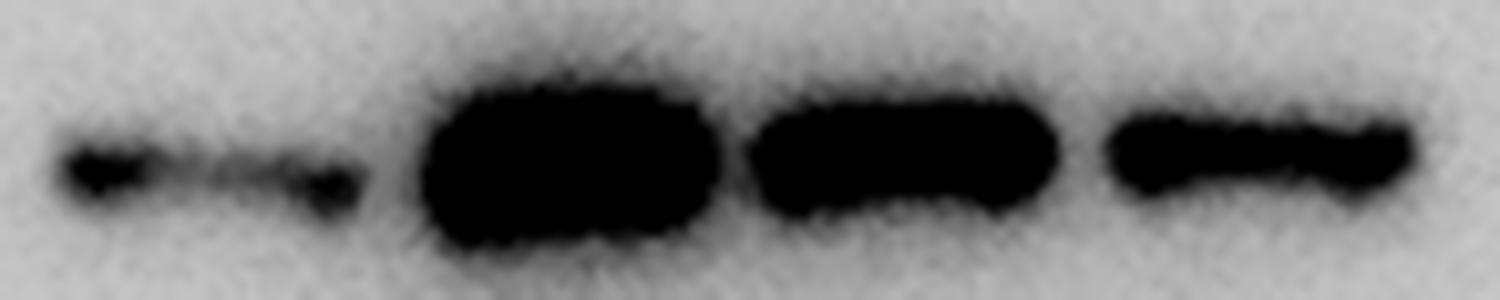

Supplement: Supplementary file 3 [file DataSheet1.ZIP › Raw Data/WB assay/Western Blotú¿Figure7ú⌐/Fig 7D/Cleaved_caspase3 (3).tif]

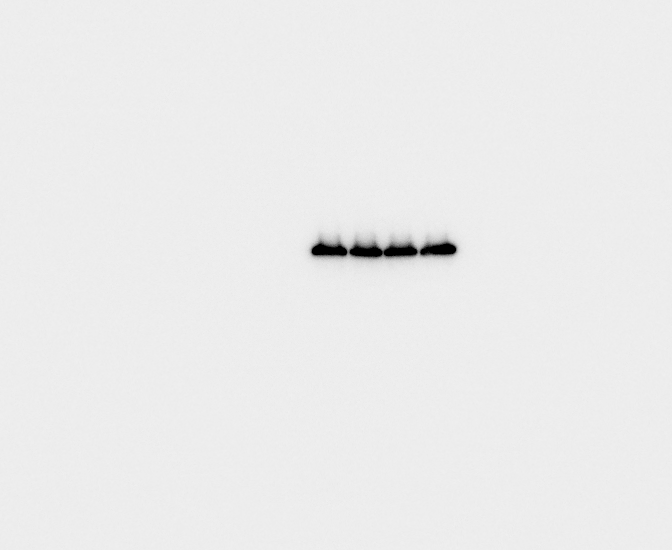

Supplement: Supplementary file 3 [file DataSheet1.ZIP › Raw Data/WB assay/Western Blotú¿Figure7ú⌐/Fig 7D/GAPDH (1).tif]

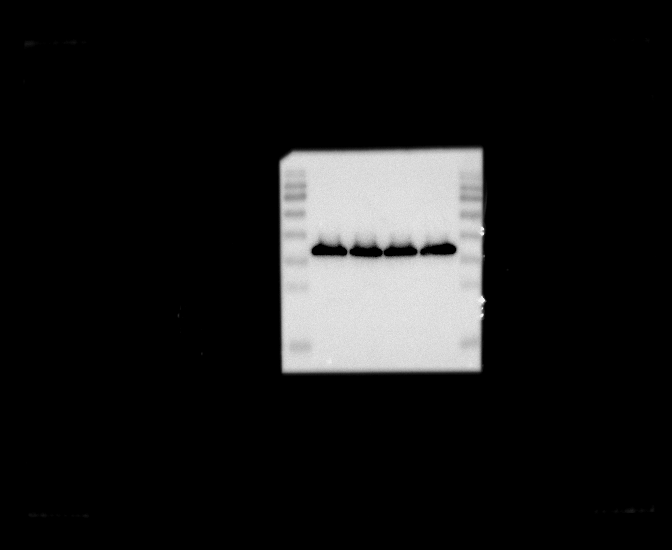

Supplement: Supplementary file 3 [file DataSheet1.ZIP › Raw Data/WB assay/Western Blotú¿Figure7ú⌐/Fig 7D/GAPDH (2).tif]

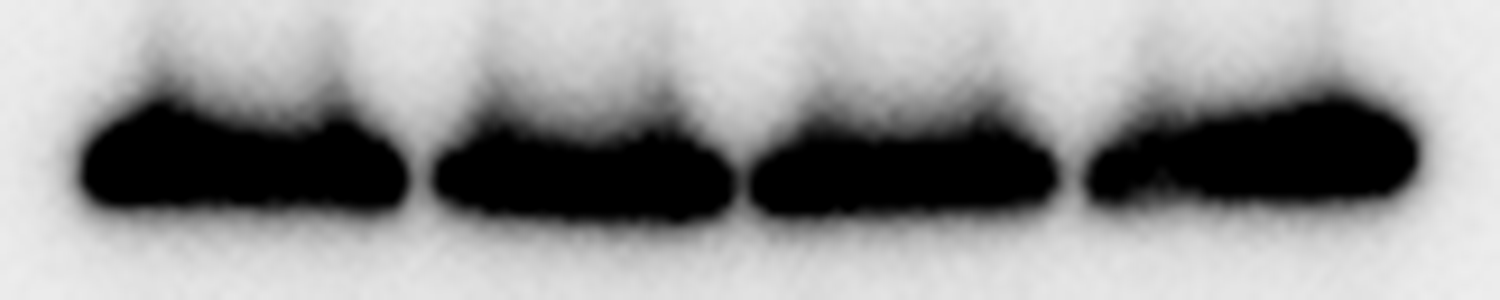

Supplement: Supplementary file 3 [file DataSheet1.ZIP › Raw Data/WB assay/Western Blotú¿Figure7ú⌐/Fig 7D/GAPDH (3).tif]

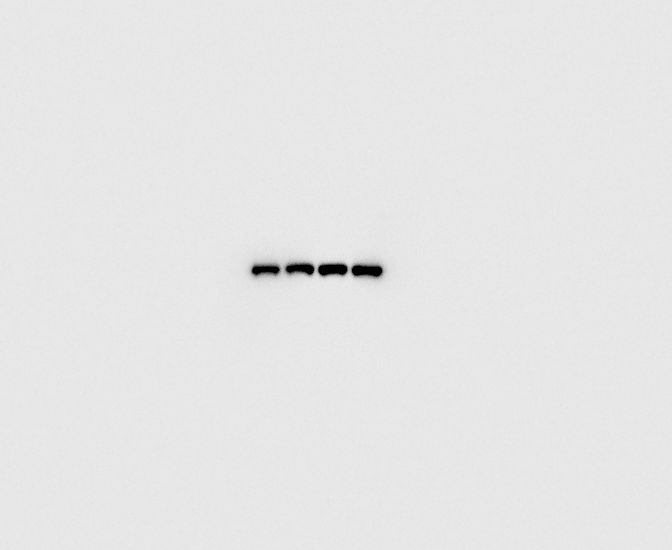

Supplement: Supplementary file 3 [file DataSheet1.ZIP › Raw Data/WB assay/Western Blotú¿Figure7ú⌐/Fig 7D/caspase3 (1).tif]

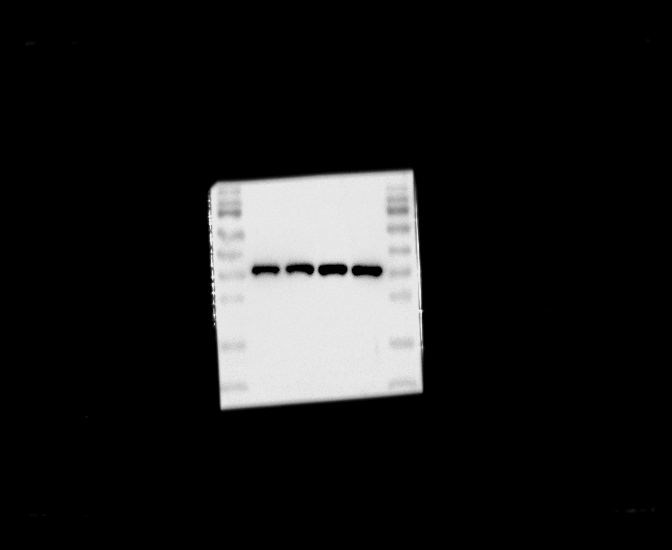

Supplement: Supplementary file 3 [file DataSheet1.ZIP › Raw Data/WB assay/Western Blotú¿Figure7ú⌐/Fig 7D/caspase3 (2).tif]

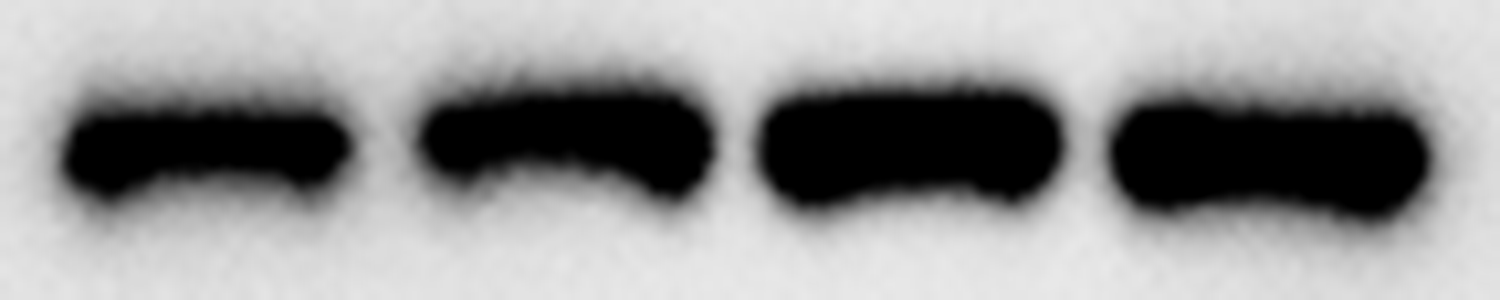

Supplement: Supplementary file 3 [file DataSheet1.ZIP › Raw Data/WB assay/Western Blotú¿Figure7ú⌐/Fig 7D/caspase3 (3).tif]

# the top 10 enriched terms of BP, CC, and MF

GO terms

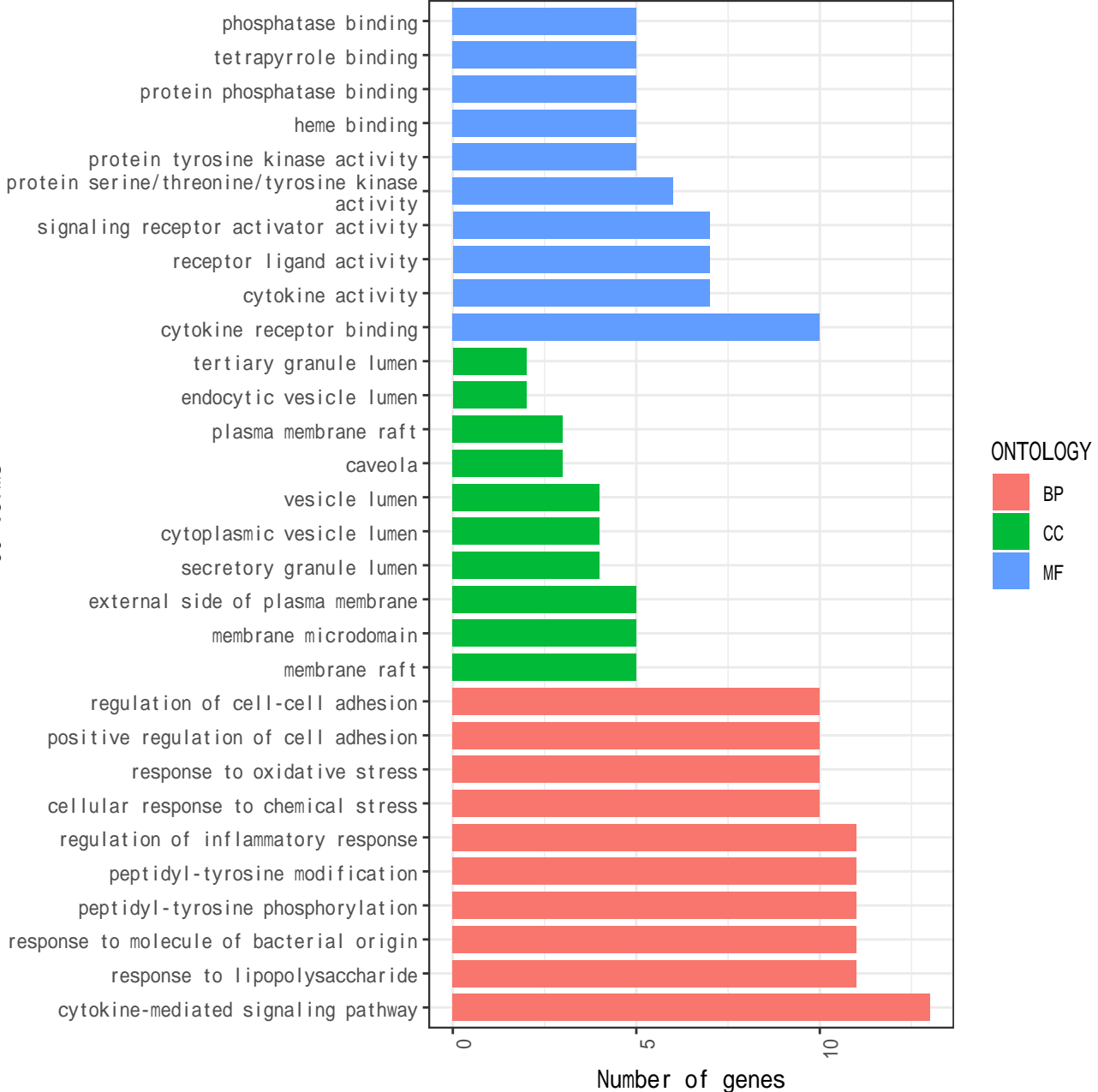

Supplement: Supplementary file 3 [file DataSheet1.ZIP › Raw Data/data_for_enrichment_analysis/go.pdf]
